# Genetic variation in seed dormancy, soil tolerance, and pH response jointly shape early establishment in *Lupinus* species

**DOI:** 10.1038/s41598-026-46460-7

**Published:** 2026-04-01

**Authors:** Ana M. Pesqueira, Ana M. González, Miriam Gallardo, Marta Santalla

**Affiliations:** Grupo de Genética del Desarrollo de Plantas (DevoLEG), Misión Biológica de Galicia-CSIC, P.O. Box 28, 36080 Pontevedra, Spain

**Keywords:** Physical dormancy, *Lupinus*, Genetic variation × environment, Soil performance, pH tolerance, Cover crops, Climate resilience, Ecology, Ecology, Plant sciences

## Abstract

The ability of seeds to overcome dormancy and establish seedlings under variable conditions determines crop establishment success in agroecosystems. Genetic diversity in dormancy-related traits and their interaction with soil and pH conditions define the ecological and agronomic limits of early establishment in *Lupinus*. We evaluated 50 accessions from three species (*L. angustifolius*, *L. luteus*, and *L. albus*) to quantify how variation in seed-coat impermeability interacts with edaphic constraints. Using three complementary experiments—standardized scarification assays, multi-soil trials, and pH-gradient tests—we assessed physical dormancy (PD), germination dynamics, and early seedling establishment performance. In *L. angustifolius*, mechanical scarification increased germination by up to 85%, and under non-scarified conditions, DDS₅₀ (days to 50% germination) varied widely among accessions (from < 10 to > 200 days). However, low-PD accessions did not consistently achieve high establishment success, indicating that dormancy traits alone do not predict establishment. Generalized linear mixed models revealed strong species × soil and species × pH interactions for emergence and establishment success (*P* < 0.001), with establishment rates varying from < 20% to > 80% among accessions and environments. Multivariate analyses (PCA and clustering) differentiated accessions with broad versus narrow establishment responses. An integrative, phenotype-based selection index combining establishment success, seedling length, and performance stability under pH gradients identified *L. albus* and *L. luteus* accessions with consistent performance across soils, and a subset of *L. angustifolius* accessions performing well under alkaline conditions. Our findings demonstrate that seed dormancy, soil compatibility, and pH tolerance act as complementary filters, and that integrative phenotyping frameworks can guide genetic selection of resilient *Lupinus* cover crops for Mediterranean orchards and other perennial systems under climate variability.

**Supplementary Information:**

The online version contains supplementary material available at 10.1038/s41598-026-46460-7.

## Introduction

Seed germination and seedling emergence are critical stages that determine plant establishment in variable environments. Seed dormancy regulates this process by synchronizing germination with favorable conditions and contributing to long-term persistence^1,2^. In legumes, dormancy is most often expressed as physical dormancy (PD), caused by an impermeable seed coat that restricts water uptake^3–5^. This barrier enables seeds to persist in soil seed banks and buffer against environmental variability, but in agricultural systems, it can hinder uniform emergence and reduce productivity^1^. PD release occurs when the seed coat is disrupted by scarification, temperature fluctuations, or microbial activity^1,6^. Genetic and anatomical differences in seed coat structure strongly influence the rate and sensitivity of dormancy release, giving rise to heritable variation among accessions^7,8^. Responses to these cues vary among *Lupinus* accessions, reflecting differences in seed coat anatomy, domestication history, and local environmental conditions.

The genus *Lupinus* comprises more than 200 species distributed across Mediterranean-type climates and cultivated for food, feed, green manure, and ecosystem services. In southern Europe, *L. angustifolius* (narrow-leafed lupin), *L. luteus* (yellow lupin), and *L. albus* (white lupin) are of major agronomic importance. These species differ in phenology, seed composition, and establishment capacity under contrasting soil conditions. *L. angustifolius* is broadly cultivated in Mediterranean drylands and has undergone extensive breeding improvement^9^. *L. luteus* thrives in acidic, sandy, nutrient-poor soils and shows rapid early growth with high nitrogen fixation^10,11^. *L. albus* produces high biomass and tolerates low pH and phosphorus deficiency^12^. These interspecific differences stem partly from contrasting domestication trajectories: selection in *L. angustifolius* has reduced PD and enhanced synchrony, whereas *L. luteus* and *L. albus* retain wider genetic variation for dormancy and edaphic tolerance. Such diversity provides an opportunity to explore how genetic variation in dormancy and early growth traits interacts with multiple environmental filters to determine establishment success.

Previous studies on PD in *Lupinus* have mainly focused on single species under controlled conditions. In *L. angustifolius*, substantial variation in seed coat impermeability and its genetic basis has been reported^13,14^, while in *L. albus* dormancy has been progressively reduced through domestication, with modern cultivars showing lower PD than wild accessions^15^. By contrast, information on *L. luteus* dormancy remains scarce despite its agronomic relevance in acidic and nutrient-poor soils. Importantly, the extent to which this genetic variation interacts with soil physicochemical properties or pH to influence early establishment has not been addressed. Moreover, few studies have jointly examined dormancy, germination, and early seedling establishment performance across multiple *Lupinus* species, and even fewer have assessed how these processes are shaped by contrasting soil properties or pH. This lack of integrative multi-species analyses limits our capacity to predict establishment in heterogeneous Mediterranean agroecosystems and underscores the need for a genetic variation × environment (G × E) approach linking dormancy mechanisms to soil- and pH-mediated establishment responses.

Soil properties—including pH, texture, organic matter, and nutrient balance—are major determinants of germination, seedling growth, and early establishment performance^16^. In Mediterranean systems, these factors often vary at short spatial scales, creating strong constraints for uniform crop establishment. Legume cover crops improve soil structure, water infiltration, and nutrient retention, while also fixing atmospheric nitrogen and promoting beneficial microbial associations such as rhizobia and mycorrhizae^17–19^. In *Lupinus*, edaphic factors interact with dormancy traits to shape establishment success. For example, both *L. luteus* and *L. albus* are commonly associated with acidic soils but differ in phosphorus acquisition strategies: *L. luteus* relies on specialized root traits to mobilize P in nutrient-poor substrates, while *L. albus* exhibits broader tolerance to P deficiency^11,12^. Soil reaction (pH) further acts as a primary environmental filter, affecting nutrient availability, microbial compatibility, and early organ allocation. Understanding these interactions is critical for selecting *Lupinus* accessions that combine low dormancy with soil- and pH-specific establishment performance, thereby enhancing their function as multifunctional cover crops in Mediterranean orchard systems.

Mediterranean orchards—including citrus, olive, almond, and vineyards—are particularly prone to soil degradation due to permanent planting and associated management practices. These systems face erosion, nutrient depletion, low organic matter, and reduced water infiltration, problems exacerbated by climate variability with more frequent droughts and intense rainfall events. In addition to improving soil health, winter legume cover crops can provide ecological services by sustaining arthropod diversity and supporting biological pest control. The benefits of *Lupinus* cover crops in these systems depend on selecting accessions with genetic and physiological traits matched to local soils and capable of establishing uniformly under variable climates^20,21^. Although citrus soils were used in this study, the framework developed applies broadly to Mediterranean perennial systems where soil heterogeneity and climate variability constrain establishment.

Here, we integrated phenotypic assessment of physical dormancy (PD) with multi-soil and pH-gradient assays for early establishment in three agronomically relevant *Lupinus* species (*L. angustifolius*, *L. luteus*, and *L. albus*). We expected that accessions would differ widely in physical dormancy and dormancy-release dynamics, that early establishment would be strongly filtered by soil physicochemical properties in a species- and accession-dependent manner, and that substrate pH would constrain early seedling growth and establishment, with alkaline conditions acting as a major filter for a subset of accessions. Our objectives were to: (i) quantify and classify phenotypic variation in PD among accessions of *L. angustifolius* under scarified and non-scarified conditions; (ii) compare germination, cotyledon emergence, and establishment success across contrasting soils in all three species; and (iii) assess the effect of substrate pH on seedling growth and establishment performance using the same multi-species panel. Each experiment was designed to isolate one major environmental filter (physical dormancy, soil physicochemical properties, or substrate pH), enabling their independent evaluation and joint interpretation within a unified framework of early establishment. This framework facilitates the identification of *Lupinus* ideotypes combining heritable dormancy release, edaphic tolerance, and pH responsiveness—traits essential for resilient cover crops in Mediterranean perennial systems.

## Material and methods

### Plant material

**Fig. 1:**
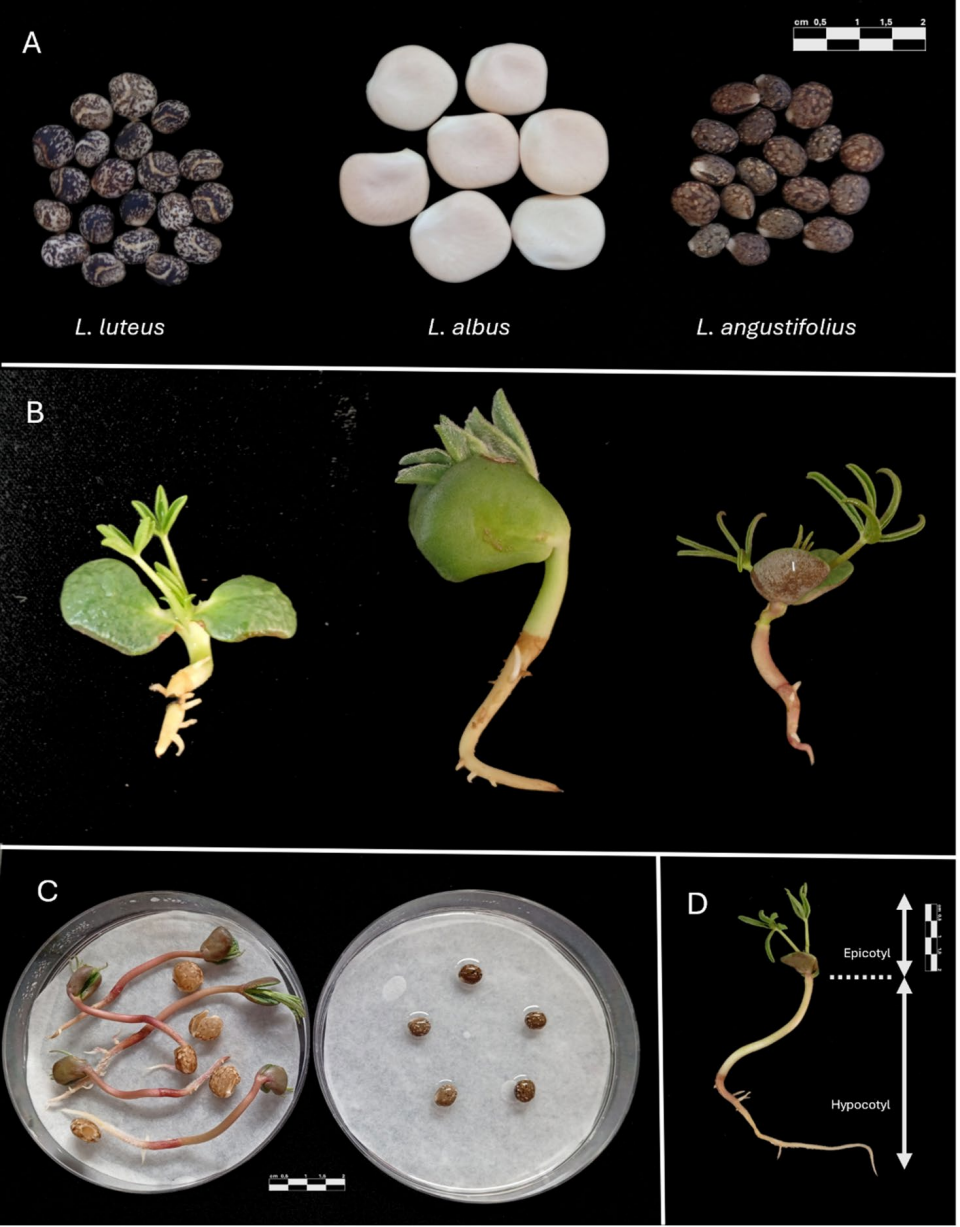
Seed and seedling morphology of three *Lupinus* species and illustration of measured traits. (**A**) Seeds of *L. luteus*, *L. albus*, and *L. angustifolius* showing interspecific differences in size, colour, and seed-coat appearance (scale bar = 2 cm). (**B**) Representative seedlings of each species at early establishment stages (left to right: *L. luteus*, *L. albus*, *L. angustifolius*). (**C**) Germination assay of *L. angustifolius* showing scarified (left) and non-scarified (right) seeds after 7 days of incubation, illustrating differences in physical dormancy release (scale bar = 1 cm). (**D**) Example of seedling indicating measured shoot organs: hypocotyl and epicotyl lengths (scale bar = 1 cm).

A total of 50 *Lupinus* accessions, including wild and cultivated material from three species—*L. angustifolius* (n = 17), *L. luteus* (n = 16), and *L. albus* (n = 17)—were used across experiments (Fig. 1; Table S1). In this study, each accession was considered a reference population and the primary genetic unit of analysis. Seeds were obtained from recognized genebanks and breeding programs (Centro de Investigaciones Científicas y Tecnológicas de Extremadura, CICYTEX; Centro de Recursos Fitogenéticos (CRF, INIA-CSIC, Madrid); and MBG-CSIC). Passport codes, geographic origin, and domestication status (wild, landrace, or cultivar) are provided in Table S1. Seed purity and viability were verified following ISTA rules to ensure experimental reproducibility and to minimize maternal or lot effects on dormancy expression and seedling emergence^22,23^. Species identification was based on passport data and genebank records associated with each accession. All plant materials correspond to conserved germplasm from institutional collections and are traceable through the accession codes reported in Table S1. No voucher specimens were newly generated for this study. No field collection of plant material was carried out in this study. The use of plant material complied with relevant institutional and national regulations governing germplasm conservation and use.

### Experimental design and data collection

Three complementary experiments were conducted under controlled conditions to evaluate the effects of seed-coat integrity, soil environment, and substrate pH on seed germination and early seedling establishment performance in *Lupinus* spp. All trials followed a randomized complete block design (RCBD) adapted to each experimental context and were performed under controlled growth-chamber or greenhouse conditions, as appropriate. Each experiment focused on a single environmental filter while holding the others constant, allowing direct comparison among experiments and integration within a unified conceptual framework. Unless otherwise stated, seeds were the observational units, and accession was the primary genetic unit of inference.

*Experiment 1—Seed coat scarification (physical dormancy).* Sixteen of the 17 *L. angustifolius* accessions were evaluated for physical dormancy (PD) and germination dynamics in growth chamber. Seeds were scarified using 60–80 grit sandpaper (S) or left intact (NS), surface-sterilized (10% NaOCl for 15 min), and rinsed with sterile water. For each accession, ten seeds were assessed, with five seeds assigned to each treatment (S and NS). The experimental unit consisted of a Petri dish (60 mm) lined with Whatman No. 1 filter paper, corresponding to an accession × treatment combination. Plates were incubated at 23 ± 2 °C, 70% relative humidity, under an 8 h light/16 h dark photoperiod. Moisture was maintained with deionized water, and contaminated plates were replaced when necessary. Germination was recorded weekly for 365 days. At the end of the assay, non-germinated seeds were scarified to verify viability^22^.

The following variables were calculated at the accession × treatment level using standard seed physiology methodologies^3,8,22^: final germination percentage (FGP, defined as the percentage of seeds germinated per Petri dish), mean germination time (MGT, expressed as the average number of days to germination), germination velocity index (IVG, an indicator of germination rate and synchrony), days to 50% germination (DDS₅₀, defined as the number of days required to reach 50% cumulative germination), and physical dormancy (PD; % = 100 − FGP_NS_, where FGP_NS_ is the final germination percentage of non-scarified seeds). PD therefore represents the fraction of viable seeds that failed to germinate due to seed coat impermeability. All derived variables were computed from accession × treatment-level data prior to statistical analysis.

*Experiment 2—Soil environments*. To assess the effect of contrasting soil environments on early establishment, 48 accessions from the three species were evaluated across five agricultural soils under greenhouse conditions (Table S2). The experiment followed an RCBD with three blocks. Within each block, each accession was tested in all five soils and under both scarification treatments (S and NS), with five seeds per soil × treatment combination (i.e., 5 soils × 2 treatments × 5 seeds per block). The soils spanned pH 5.6–8.4, organic matter 0.8–2.5%, and CEC 5–18 cmolc kg⁻^1^, representing a gradient of soil conditions differing in pH, organic matter, and cation-exchange capacity*.*

Soils were homogenized, sieved (4 mm), and pre-moistened before sowing in 96-cell trays. Plants were grown at 23 ± 2 °C under an 8 h light/16 h photoperiod and monitored weekly for 30 days using a 0–5 ordinal establishment scale (0 = no change, 1 = seed coat rupture, 2 = radicle emergence, 3 = cotyledon emergence, 4 = arrested growth or fungal damage, 5 = fully established seedling). The following variables were calculated at the accession × soil × treatment level: germination success (proportion of seeds reaching score ≥ 1), cotyledon emergence (score ≥ 3), and successful establishment (score = 5). These thresholds were aligned with Experiment 3 to facilitate cross-experiment comparisons.

*Experiment 3—Substrate pH gradient treatment.* Forty-eight accessions (Table S1) were evaluated under three substrate pH conditions—acidic (pH 5.5), neutral (pH 7.0), and alkaline (pH 8.5)—at two time points (7 and 14 days after sowing, DAS7 and DAS14). DAS was included as a developmental factor to distinguish temporal from pH effects. The experiment followed a randomized complete block design, with three independent replicates per accession × pH combination. The experimental unit was a 90 mm Petri dish (accession × pH × replicate), containing five seeds. All seeds were scarified, surface-sterilized, and rinsed, then placed in Petri dishes lined with Whatman No. 1 filter paper and moistened with 3 mL of Hoagland nutrient solution adjusted to the target pH level. Plates were sealed with parafilm and incubated at 23 ± 2 °C under an 8 h light/16 h dark photoperiod. Acidification or alkalinization was performed to reach the target pH levels (5.5, 7.0, and 8.5) using diluted nitric acid and sodium hydroxide as pH-adjusting agents. These reagents were used solely to adjust the final pH of the Hoagland solution and were not treated as separate experimental factors. Solutions were renewed every 2–3 days to maintain hydration and pH stability.

The following variables were calculated per replicate: germination success (binary; emergence ≥ 1), hypocotyl and epicotyl length (mm), total seedling length (mm) = hypocotyl + epicotyl, establishment success (binary; total length ≥ 30 mm), hypocotyl-to-epicotyl ratio = Hypocotyl / (Epicotyl + 1), where a constant of 1 mm was added for numerical stability in seedlings with measured but non-elongated epicotyls, and hypocotyl allocation = Hypocotyl / Total length. Seedlings lacking either hypocotyl or epicotyl measurements (n = 883) were excluded from growth and allocation analyses. The number of valid observations per pH × DAS combination is provided in Table S3. An integrated selection index was computed at DAS14 based on three equally weighted components derived from the substrate pH experiment (Experiment 3). These components included: (i) mean establishment success across pH 5.5 and 7.0, calculated as the average proportion of seedlings reaching a total length ≥ 30 mm at each pH level; (ii) mean total seedling length across pH 5.5 and 7.0, expressed as the average hypocotyl + epicotyl length (mm); and (iii) stability, defined as 1/(|Establishment_(pH7.0)_—Establishment_(pH5.5)_|+ 1), capturing consistency of establishment performance across contrasting pH conditions. Alkaline pH (8.5) was excluded from the stability component to avoid bias arising from inhibitory conditions. Prior to index calculation, each component was rescaled to the [0, 1] range to ensure equal contribution of establishment success, seedling growth, and performance stability. The integrated selection index (ISI) was calculated as the mean of the three normalized components and was used to rank accessions and identify top-performing accessions under non-limiting pH conditions. Although the index was derived exclusively from Experiment 3, responses from the soil treatment (Experiment 2) and dormancy behaviour in *L. angustifolius* (Experiment 1) were analyzed independently and interpreted jointly at a conceptual level, following standard approaches in multi-environment legume trials^24,25^.

### Data analysis

Statistical analyses were designed to evaluate species-level responses and accession-level performance across the three experimental factors: seed-coat treatment (as a primary factor in Experiment 1 and as a secondary factor in Experiment 2), soil environment (Experiment 2), and substrate pH (Experiment 3). Continuous germination- and growth-related variables were analyzed using two- or three-way ANOVAs, depending on the experimental design, followed by Tukey’s HSD or Dunnett’s tests for multiple comparisons. Polynomial regression was applied to model trait responses along the pH gradient (Experiment 3), and logit-transformed allocation ratios were tested using linear regression. Long-term germination dynamics (Experiment 1) were assessed using Kaplan–Meier survival curves and log-rank tests.

Multivariate analyses included principal component analysis (PCA) and hierarchical clustering (Euclidean distance, Ward’s method). Cluster robustness was evaluated by multiscale bootstrap resampling, and cohesion by the silhouette index. PCA outputs included trait loadings, variance contributions, and accession scores, with soil physicochemical properties and substrate pH incorporated as supplementary variables. Pairwise associations among traits and experiments were estimated using Spearman’s ρ with 95% bootstrap confidence intervals, and p-values were adjusted using the Benjamini–Hochberg false discovery rate (FDR) correction. Correlation matrices were visualized as heatmaps.

Linear (LMMs) and generalized (GLMMs; binomial error) mixed-effects models were applied to test the influence of species, environment, and treatment on continuous and binary traits, with accession fitted as a random intercept to account for accession-level clustering and non-independence of observations within accessions. Fixed terms were specified as Species × Soil + Scarification (Experiment 2) and Species × pH + DAS (Experiment 3). Model performance was evaluated using AIC, marginal and conditional R^2^, and residual diagnostics. Post hoc contrasts were obtained with Tukey’s adjustment. All analyses were performed in R (v. 4.4.0)^26^ using RStudio (version 2024.04.2 + 764)^27^. Multiscale bootstrap clustering was conducted using the pvclust package^28^.

## Results

### Dormancy release and germination dynamics in response to scarification

**Fig. 2:**
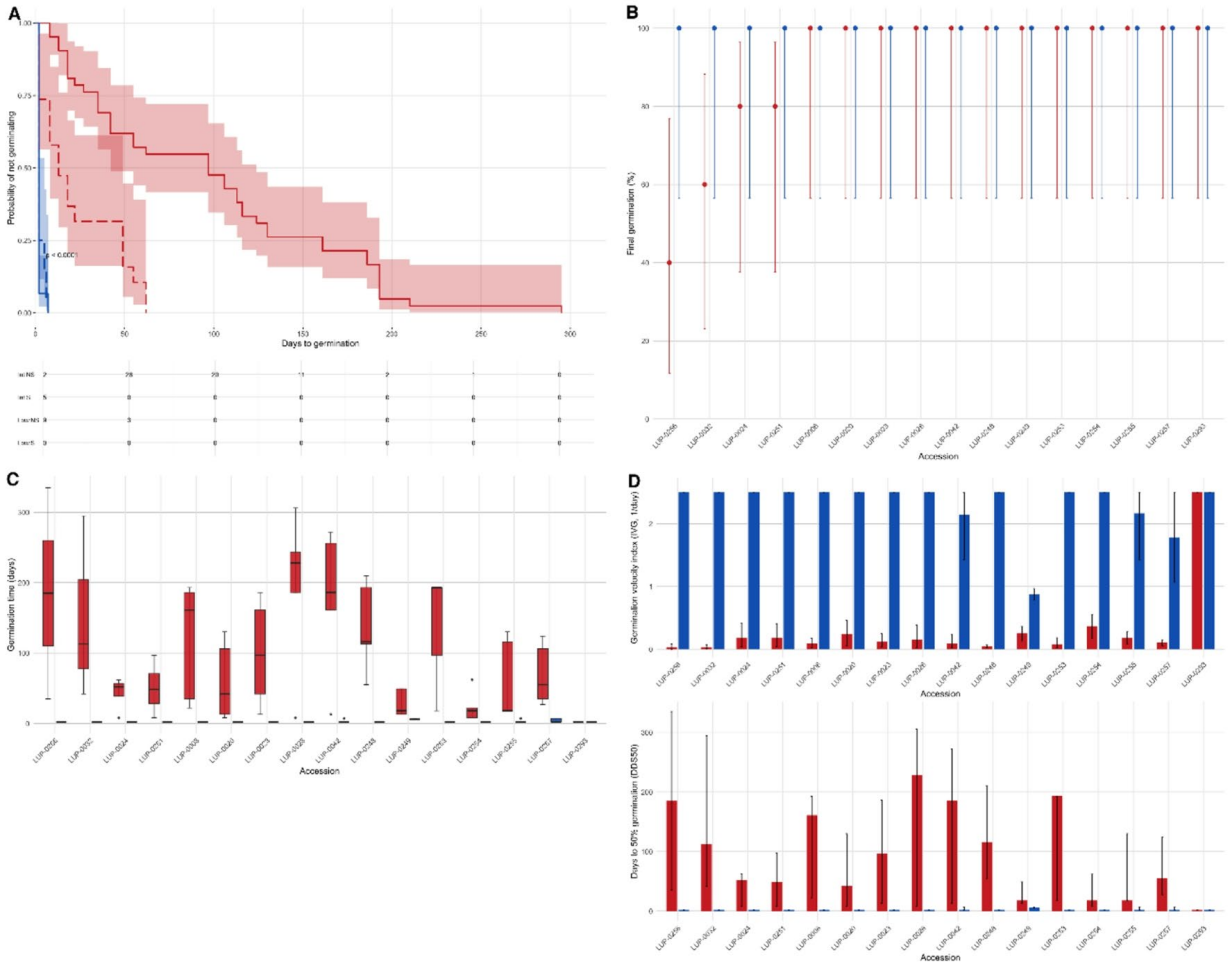
Effects of seed-coat scarification on germination dynamics in 16 *L. angustifolius* accessions under non-scarified (NS) and scarified (S) treatments. (**A**) Kaplan–Meier curves showing time to germination (days) for NS (red) and S (blue) seeds in the intermediate-dormancy class (solid lines) and the low-dormancy class (dashed lines); shaded areas indicate 95% confidence intervals (CIs). Risk tables indicate the number of seeds remaining ungerminated (“at risk”) at each time point. Groups differed significantly (log-rank test, *p* < 0.001). (**B**) Final germination percentage (FGP) by accession and treatment (means ± 95% binomial CIs); ordered by FGP under NS to highlight accessions with greater response to scarification. (**C**) Germination time (days) by accession and treatment (boxplots showing median, interquartile range, and whiskers = 1.5 × IQR). (**D**) Germination-speed and synchrony indices (IVG and DDS₅₀). Two-way ANOVA showed significant effects of accession, treatment, and their interaction (p < 0.001). See Table S4 or ANOVA results.

In *Lupinus angustifolius*, seed coat scarification produced a clear and consistent effect on the release of physical dormancy (PD) and subsequent germination dynamics, representing the first environmental filter acting on early establishment. Germination curves showed strong treatment effects of scarification on germination timing, particularly within low- and intermediate-dormancy classes (Fig. 2A). Dormancy classes (low, intermediate, high) were assigned based on the phenotypic classification derived from the non-scarified germination profiles integrating FGP, MGT, DDS₅₀, and IVG (Table S5). Scarified seeds (S) germinated rapidly, typically within ~ 20 days, whereas non-scarified seeds (NS) exhibited delayed and often incomplete germination, with several accessions remaining ungerminated after 300 days. Thus, scarification consistently accelerated germination and increased final germination proportion across dormancy classes (log-rank test, *p* < 0.001). This result reflects the role of seed-coat impermeability in controlling water uptake.

At the accession level, final germination percentage (FGP) was consistently high under scarification (S), approaching complete germination for all accessions, whereas under non-scarified (NS) conditions a subset of accessions (LUP-0024, LUP-0251, LUP-0032, LUP-0256) showed reduced FGP and responded strongly to scarification, shifting to near-complete germination after S (Fig. 2B). Mean germination time (MGT) showed wide variation among accessions under NS conditions, ranging from < 10 days in fast-germinating accessions to > 60 days in highly dormant ones, but was consistently reduced after scarification (Fig. 2C). Two-way ANOVA detected significant effects of accession, treatment, and their interaction for all germination-timing indices (MGT, IVG, DDS₅₀; p < 0.001; Table S4, confirming the strong and consistent impact of scarification on dormancy release and germination rate. These interactions indicate that dormancy intensity behaves as a quantitative, accession-dependent trait rather than a binary attribute, consistent with continuous variation in seed-coat impermeability. Indices describing germination speed and synchrony further supported these patterns: scarification reduced DDS₅₀ and increased IVG across accessions, with consistently lower variance under S conditions (Fig. 2D). Under NS conditions, DDS₅₀ ranged from < 10 days in low-dormancy accessions to > 200 days in highly dormant ones, while values approached zero following scarification. Overall, Experiment 1 demonstrated that physical dormancy in *L. angustifolius* operates along a continuous spectrum among accessions, and that scarification effectively collapses this gradient into a comparatively uniform post-dormancy germination response.

**Fig. 3:**
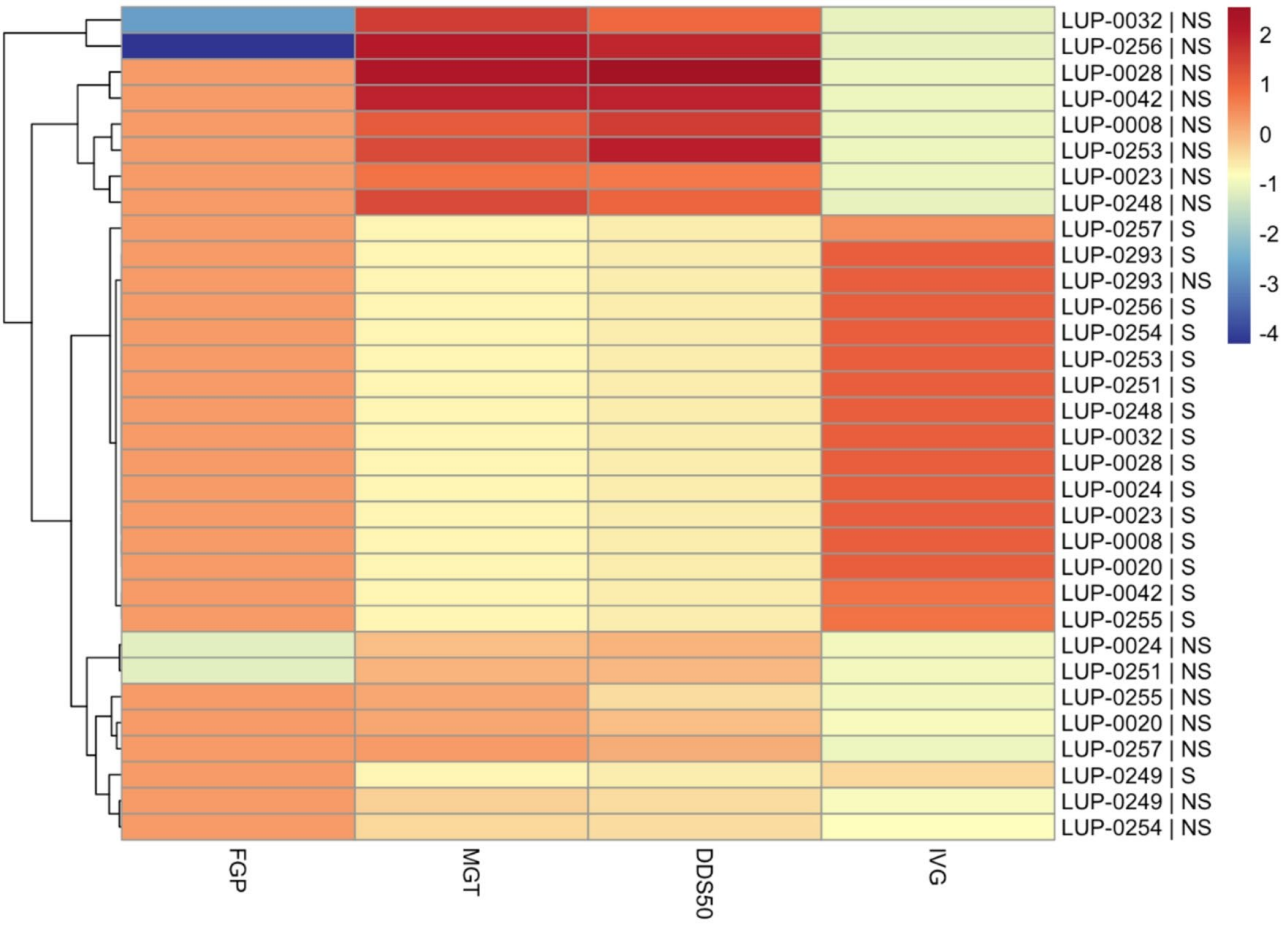
Inter‑accession variability and phenotypic profiling of dormancy-related germination responses under non‑scarified (NS) and scarified (S) conditions. Heatmap of standardized accession × treatment means (Z-scores) for four germination parameters: final germination percentage (FGP), mean germination time (MGT), days to 50% germination (DDS₅₀), and germination velocity index (IVG). Rows represent accession x treatment combinations (NS or S), and columns represent traits. Hierarchical clustering (Euclidean distance, Ward.D2 method) identifies groups of accessions with similar baseline dormancy-related responses and phenotypic responsiveness to scarification. See Table S5 for accession-level means and PCA scores.

Patterns of FGP revealed wide inter-accession variation under NS conditions, indirectly reflecting physical dormancy (PD = 100 − FGP_NS_) (Fig. 3). Four accessions (LUP-0024, LUP-0032, LUP-0251, LUP-0256) showed measurable dormancy, which was fully released by scarification. Standardized profiles combining FGP, MGT, DDS₅₀, and IVG highlighted contrasting phenotypic responses to scarification, with two accessions—LUP-0032 and LUP-0256—displaying the full NS signature (low FGP and IVG, high MGT and DDS₅₀) and a shift to rapid, synchronous germination after treatment. By contrast, LUP-0024 and LUP-0251 showed only moderate dormancy and smaller timing shifts, approaching the response patterns observed in low-dormancy accessions that germinated rapidly even without treatment (Fig. 3; Table S5). Pairwise correlations confirmed strong associations among time-related indices (MGT and DDS₅₀) and their inverse relationship with IVG, whereas FGP displayed weaker and more variable correlations across treatments (Fig. S1; Table S6).

Principal component analysis of the NS dataset provided an integrated view of inter-accession phenotypic patterns (Fig. S2; Table S7). PC1 captured the main dormancy-related axis, contrasting time-related indices with vigor and final success, and explained approximately 65% of the total variance. PC2 represented secondary variation, mainly distinguishing residual differences between FGP and IVG, bringing the cumulative explained variance to 87%. Low-dormancy accessions clustered distinctly, whereas intermediate- and high-dormancy groups were distributed along a continuous gradient of germination responses (Table S5). PCA was not performed for the S treatment, as germination was nearly uniform across accessions.

In summary, Experiment 1 uncovered marked phenotypic variation among *L. angustifolius* accessions in physical dormancy and germination dynamics. Survival analysis, germination metrics, and multivariate ordination enabled the classification of accessions into three dormancy response groups (low, intermediate, high), while revealing a continuous gradient from rapid, synchronized germination to delayed and incomplete emergence.

### Environmental modulation of early emergence: species–soil interactions

**Fig. 4:**
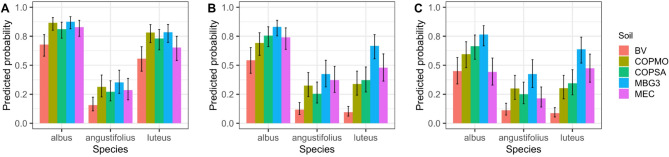
Predicted probabilities of germination, cotyledon emergence, and early establishment performance across species and soils. (**A**) Predicted probability of germination, (**B**) cotyledon emergence, and (**C**) overall early establishment probability. GLMM-derived estimates are shown for each species (*L. albus*, *L. angustifolius*, *L. luteus*), averaged across scarification treatments (non-scarified, NS; scarified, S). Error bars represent 95% confidence intervals. Soils include BV, COPMO, COPSA, MBG3, and MEC. See Table 1 for physicochemical properties.

A second experiment across five contrasting soils evaluated early seedling establishment in 48 accessions from three *Lupinus* species (Tables S1, S2). Generalized linear mixed models (GLMMs; logit scale) revealed strong species–soil interactions for early emergence traits (Fig. 4). Across all species, the BV soil—despite its high fertility profile (Table 1)—showed consistently low predicted probabilities of germination, cotyledon emergence, and early establishment, indicating that nutrient availability alone did not guarantee successful establishment. This response suggests that soil physicochemical constraints, particularly elevated salinity and exchangeable K⁺ levels, can override fertility advantages.

**Table 1:** Physicochemical properties of the five experimental soils used in germination assays. Soils were selected to represent contrasting pH and nutrient status and are described here solely by their measured physicochemical characteristics.

Soil code	MEC	BV	COPSA	COPMO	MBG3
pH H₂O (1:2.5)	8.4	7.3	8.3	8.2	6.3
pH KCl (1:2.5)	7.9	7.3	7.6	7.4	5.7
Organic matter (OM, %)	1.6	7.2	2.5	3.1	5.6
Available P (ppm)^1^	21.0	187.1	12.0	44.0	54.0
Exchangeable K (ppm)^2^	172.0	1504.0	204.0	566.0	130.0
Exchangeable Mg (ppm)	206.1	696.1	184.0	312.0	96.1
CEC (cmolc kg⁻^1^)	26.7	34.5	31.6	34.9	12.9
Electrical conductivity (EC 1:5, mmho cm⁻^1^)	0.1	0.70	0.17	0.13	0.0
Ca/Mg	15.1	4.0	20.1	12.0	15.1
Ca/K	56.7	6.5	57.5	21.8	36.3
Mg/K	3.8	1.5	2.9	1.8	2.4
Key soil feature	Moderately alkaline, low OM	Very fertile, high OM, neutral	Alkaline, low OM	Alkaline, medium OM	Acidic, high OM
Texture	Loam (low OM)	Clay loam	Clay loam	Clay loam	Clay loam
Classification	Low OM, alkaline	Reference high OM	Low OM, alkaline	Medium OM, alkaline	High OM, acidic

^1^Olsen method. ^2^Extracted with NH₄Cl.

Values represent means of composite soil samples collected at each site prior to the experiments.

In contrast, the acidic, high-organic (OM) MBG3 and the alkaline, medium-OM COPMO soils supported the highest predicted emergence and early establishment performance, especially in *L. albus* and *L. luteus* (Fig. 4; Table S8). The two alkaline, low-OM soils (MEC and COPSA) imposed species-dependent constraints on emergence and early establishment: MEC was particularly limiting for *L. luteus* and *L. angustifolius*, whereas COPSA supported relatively high establishment in *L. albus*. Accordingly, observed early-establishment values under non-scarified conditions indicated that MEC could be more restrictive than BV and COPSA for *L. albus* (Table S9). This interspecific gradient mirrors their contrasting ecological niches, with *L. albus* typically associated with neutral–alkaline soils, *L. luteus* more frequently occurring in acidic substrates, and *L. angustifolius* showing intermediate phenotypic plasticity. Pairwise contrasts (*p* < 0.05) confirmed that BV performed significantly worse than MBG3 and COPMO, with COPSA also sustaining relatively high early establishment probabilities, particularly in *L. albus*. MEC showed lower and more variable performance depending on species (Table S8).

Although correlations with DDS₅₀ (Exp. 1) were not significant, *L. angustifolius* exhibited soil-specific sensitivity, suggesting that variation in dormancy-related germination traits may partially modulate early emergence responses under certain edaphic conditions. These patterns indicate species-specific soil constraints on early development. Such soil-specific constraints may reflect, for example, physicochemical stress associated with high electrical conductivity and elevated exchangeable K in BV (Table 1). To further explore these patterns, we next assessed variation among accessions in early establishment traits across soils and identified consistent performers.

**Fig. 5:**
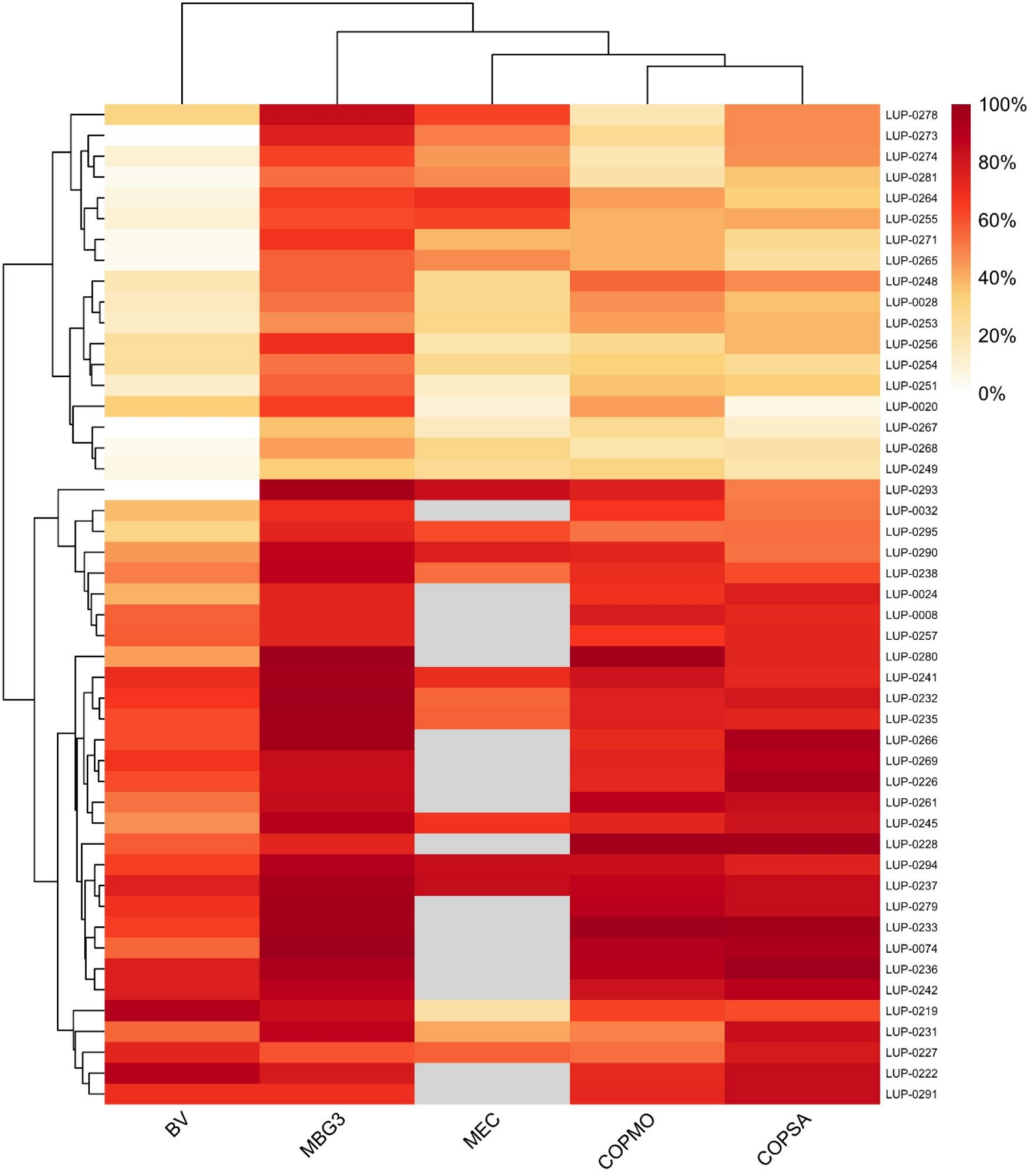
Heatmap of early seedling establishment (%) across accessions and soils. Early establishment rates (mean ± SD) were averaged across scarification treatments (NS and S) and expressed as percentages. Rows represent accessions, and columns represent soils (BV, MBG3, MEC, COPMO, COPSA). Hierarchical clustering was applied to both dimensions to group accessions with similar establishment response patterns across soils. The color scale ranges from 0% (white) to 100% (red). See Table S10 for detailed values.

Early establishment heatmaps revealed marked inter-accession variability across soils (Fig. 5). Hierarchical clustering grouped accessions by consistent performance patterns, with clear clusters of *L. albus* and *L. luteus* showing uniformly high early establishment rates (> 80%) in MBG3, COPMO, and often COPSA. In contrast, MEC showed heterogeneous responses, separating well-performing *L. albus* accessions from poorly performing *L. angustifolius* accessions, with only a few reaching > 80% (Table S10). Treatment-specific data (Table S9) confirmed that scarification enhanced early establishment across all soils but did not alter accession-level rankings. Responses across accessions strongly depended on soil type, with hotspots of high early establishment in MBG3 and COPMO/COPSA for *L. albus,* and mainly in MBG3 (and for a small subset in MEC) for *L. angustifolius*.

Multivariate analyses revealed that early seedling establishment in lupins was more strongly modulated by soil environment than by intrinsic dormancy release alone. *L. albus* and *L. luteus* achieved high early establishment rates in MBG3 and COPMO (and frequently COPSA), particularly under scarification (Table S9). In contrast, *L. angustifolius* showed greater inter-accession variability across soils, with only a subset of accessions reaching high establishment depending on soil type and germplasm type (cultivar, landrace, wild) (Fig. 5; Table S9; Table S10).

**Fig. 6:**
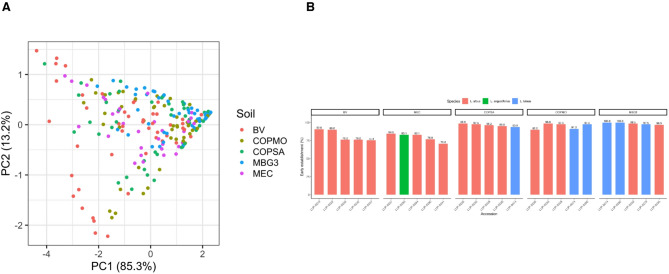
Multivariate analysis and accession-level ranking across soils. (**A**) Principal component analysis (PCA) of seedling traits (germination, cotyledon emergence, and early establishment) across accessions and soils. PC1 (85.3% of variance explained) represents overall early establishment performance, whereas PC2 (13.2%) reflects partial decoupling among establishment-related traits. (**B**) The ranking of the top five accessions per soil according to early establishment rate (%).

Principal component analysis (PCA) confirmed these soil-dependent patterns (Fig. 6A). PC1 (85.3%) represented a general axis of early seedling establishment performance, while PC2 (13.2%) captured secondary variation mainly related to cotyledon emergence and partial trait decoupling among establishment-related components. Ranking of accessions across soils (Fig. 6B) highlighted *L. albus* and *L. luteus* as top performers in the more permissive soils (MBG3, COPMO, and frequently COPSA), whereas top-ranked accessions in MEC were dominated by *L. albus*, with only occasional *L. angustifolius* accessions among the highest performers (Table S12).

**Fig. 7:**
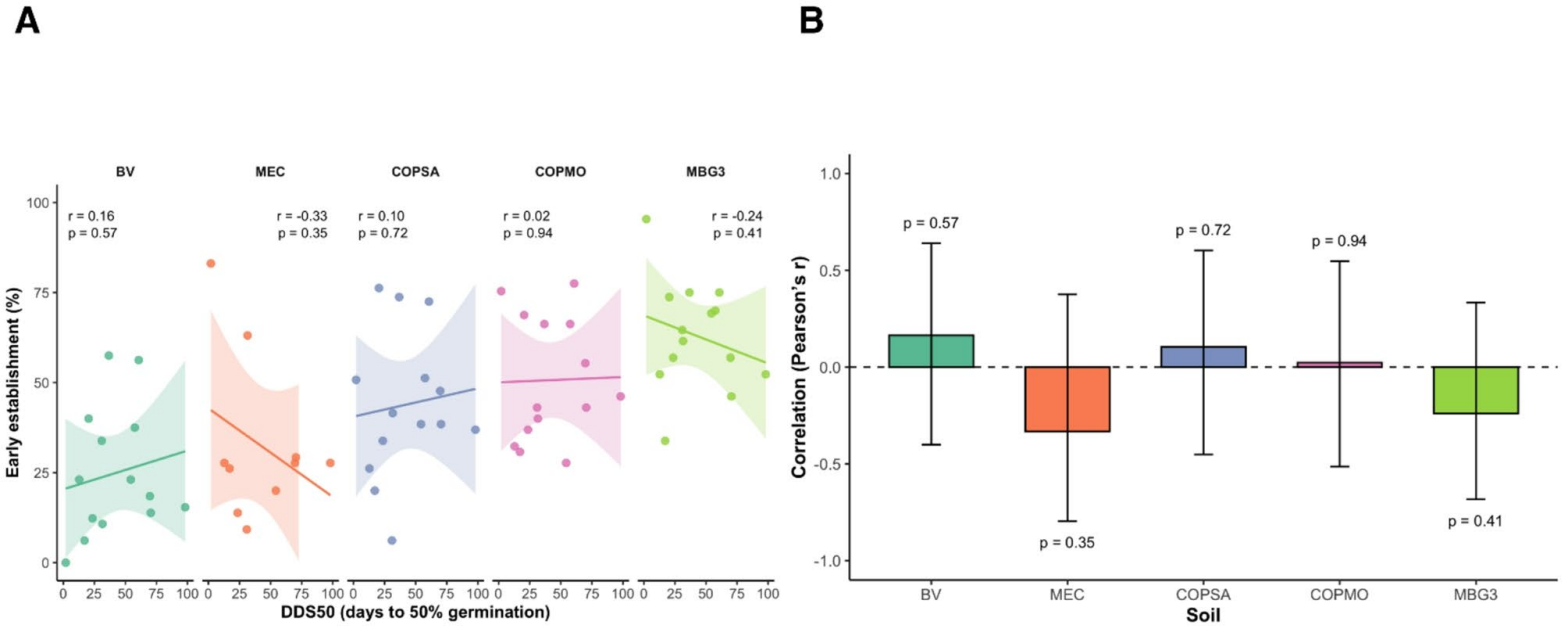
Relationship between dormancy (DDS_50_) and early seedling establishment in *L. angustifolius* across soils. (**A**) Scatterplots of DDS_50_ (days to 50% germination under controlled conditions) versus early establishment (%), with linear regression lines and 95% confidence intervals. Points represent accessions. (**B**) Pearson’s correlation coefficients (r) with 95% confidence intervals by soil. No significant correlation was detected in any soil (all p > 0.3). See Table S13 for numerical results.

To evaluate whether intrinsic dormancy release could predict establishment performance under contrasting soil conditions, we tested correlations between DDS₅₀ and early seedling establishment in *L. angustifolius* (Fig. 7; Table S13). Pearson’s r values were low and non-significant across all soils (p > 0.3), indicating that variation in DDS₅₀ did not explain differences in establishment success under these conditions. Thus, soil type operated as an environmental filter shaping early establishment independently of intrinsic dormancy release. These findings highlight the value of controlled assays for disentangling species–soil interactions.

### Effects of substrate pH and developmental time on germination, seedling establishment and growth dynamics

**Fig. 8:**
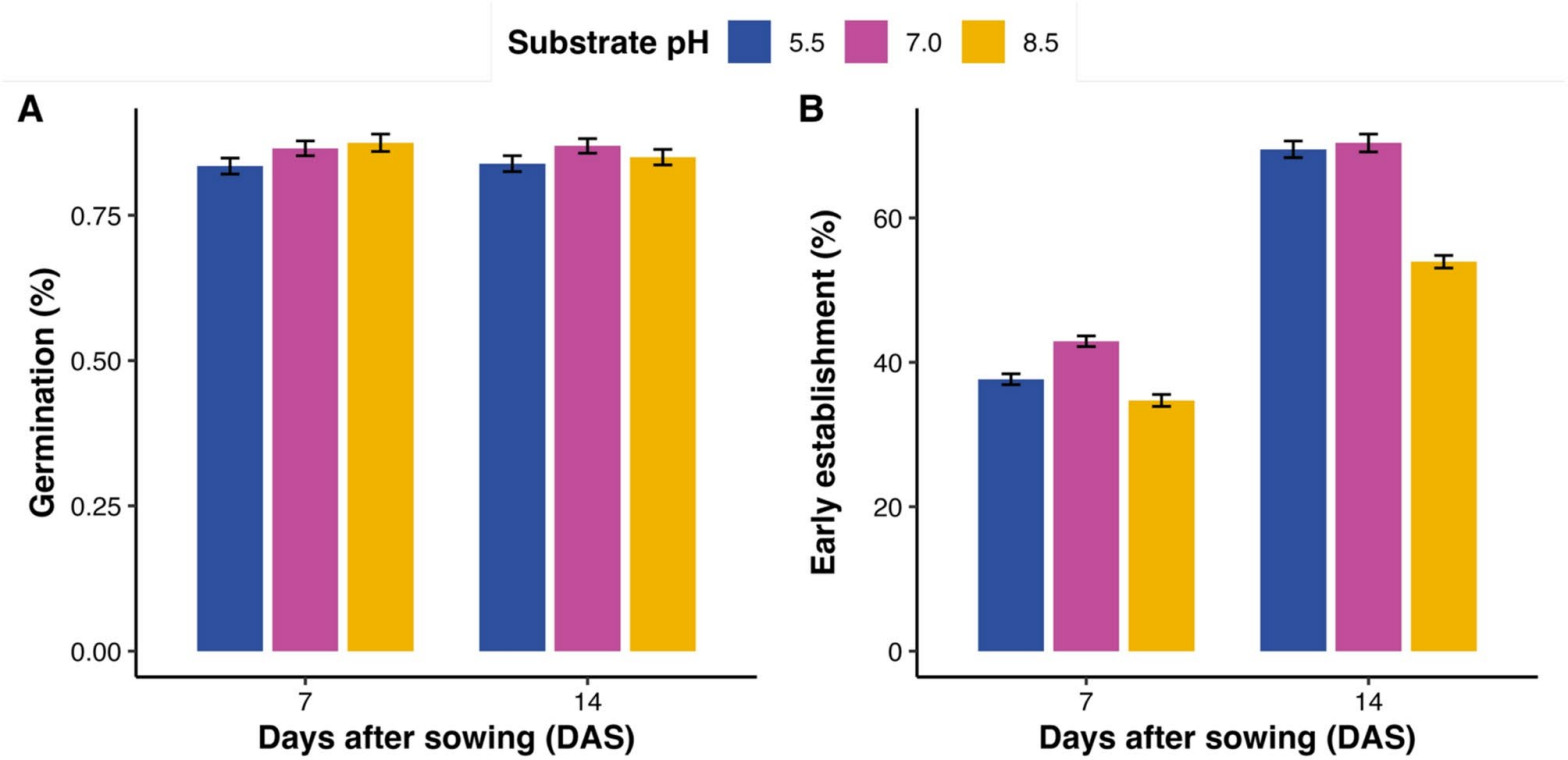
Germination and early establishment (%) of three lupin species (*L. albus*, *L. luteus*, and *L. angustifolius*) across substrate pH levels (5.5, 7.0, 8.5) at two incubation times (7 and 14 days after sowing; DAS). (**A**) Germination (%). (**B**) Early establishment (%; proportion of seedlings ≥ 30 mm). Bars show observed means ± SE, pooled across species. Results of generalized linear mixed models (GLMMs) for the effects of pH, incubation time, and their interaction are presented in Table 2, and post hoc contrasts are provided in Table S14.

To isolate the chemical component of soil effects, we assessed how substrate pH influences germination, early seedling establishment and growth in *Lupinus* through a controlled experiment using three pH levels (5.5, 7.0, and 8.5) and two incubation times (7 and 14 days after sowing, DAS). GLMM analyses detected significant effects of pH, DAS, and their interaction on germination and early seedling establishment (Fig. 8A–B; Table 2; Table S14a). At DAS14, germination did not differ significantly among pH levels (Fig. 8A; TableS14a). By contrast, early seedling establishment, defined here as the proportion of seedlings reaching ≥ 30 mm at each time point, was highest at pH 5.5 and declined at pH 8.5 in all species, with *L. luteus* exhibiting the steepest reduction (Fig. 8B; Fig. S3). Kaplan–Meier survival curves confirmed delayed emergence under alkaline conditions (pH 8.5) (Fig.S4). Overall, while final germination percentages changed little, alkaline conditions consistently delayed emergence and reduced early seedling establishment, whereas neutral and mildly acidic pH favored more balanced development.

**Table 2:** GLMM summary for germination and early establishment under substrate pH and days after sowing (DAS) treatments.

Predictor	Germination—OR (95% CI)	*p-*value	Early establishment—OR (95% CI)	*p-*value
Intercept (pH 5.5, DAS 7)	**5.05** (4.17–6.18)	< 0.0001 ***	1.04 (0.89–1.21)	0.640
pH 7.0 vs 5.5	1.27 (0.95–1.70)	0.105	**1.81** (1.46–2.25)	< 0.0001 ***
pH 8.5 vs 5.5	1.09 (0.82–1.45)	0.561	0.86 (0.68–1.09)	0.222
DAS 14 vs 7	1.03 (0.78–1.36)	0.831	**3.17** (2.52–4.00)	< 0.0001 ***
pH 7.0 × DAS 14	1.01 (0.67–1.52)	0.978	**0.64** (0.46–0.89)	0.007 **
pH 8.5 × DAS 14	–	–	–	–

Odds ratios (OR) with 95% confidence interval (CI) and p-values are shown for fixed effects in binomial generalized linear mixed models (GLMMs). Reference levels are pH 5.5 and DAS 7. DAS = days after sowing under substrate incubation (7 or 14 days). Germination and early establishment were modelled as binary outcomes evaluated at each time point (DAS 7 and DAS 14). Early establishment success corresponds to the proportion of seedlings ≥ 30 mm. OR > 1 indicates a relative increase in probability; OR < 1 indicates a decrease. The interaction term for pH 8.5 × DAS 14 could not be estimated due to lack of data (denoted by “–”). Significance codes: *** *p* < 0.001; ** *p* < 0.01; * *p* < 0.05.

**Fig. 9:**
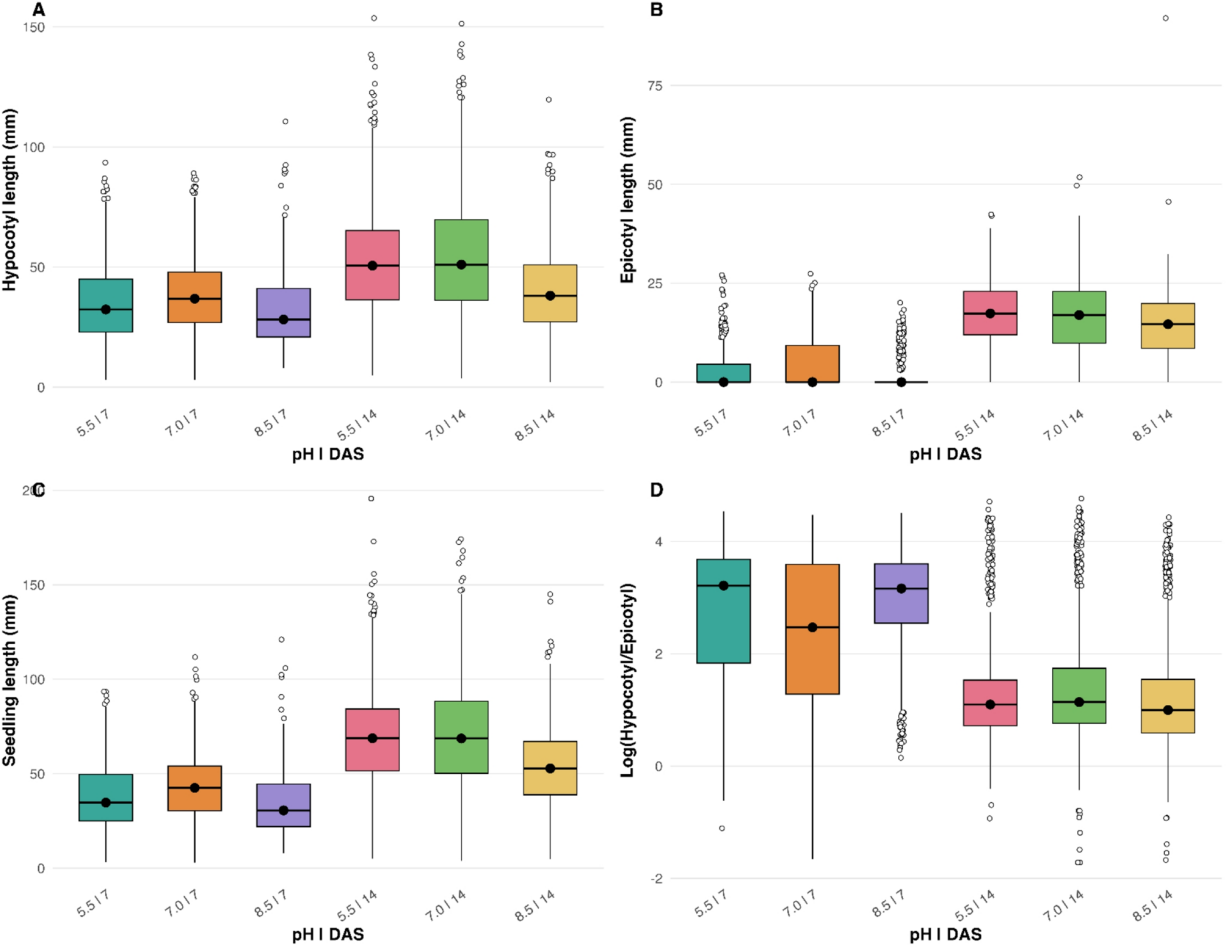
Seedling growth and allocation traits across substrate pH levels and time after sowing. (**A**) Hypocotyl length, (**B**) epicotyl length, (**C**) total seedling length, and (**D**) log-transformed hypocotyl-to-epicotyl ratio measured at 7 and 14 days after sowing (DAS) under pH 5.5, 7.0, and 8.5. Each panel shows raw trait distributions across the six pH × DAS treatment combinations. Boxplots display medians (black dots), interquartile ranges, whiskers, and outliers. In panel B, epicotyl growth was strongly constrained under specific conditions (e.g., pH 8.5 at 7 DAS), resulting in compressed distributions near zero. Panel D shows the log-transformed hypocotyl-to-epicotyl ratio for visualization, whereas statistical analyses were performed on untransformed values. See Table 3 and Table S14 for ANOVA and post hoc test results.

Across seedling organs, time after sowing was the dominant driver of elongation (Fig. 9; Table 3). Between 7 and 14 DAS, seedlings increased by ~ 14 mm in hypocotyl, ~ 12 mm in epicotyl, and ~ 26 mm in total length (Tukey *p* < 0.0001). Accordingly, DAS accounted for the largest *F*-values across traits (Table 3), reflected by the upward shift of boxplots in Fig. 9A–C. Substrate pH further modulated these gains and interacted with time. At 7 DAS, elongation ranked 7.0 > 5.5 > 8.5 (*p* ≤ 0.002), whereas at 14 DAS, both 5.5 and 7.0 exceeded 8.5 (*p* < 0.0001; Table S14b). For the epicotyl, the interaction was again significant: elongation at 7 DAS peaked at pH 7.0, but by 14 DAS it was markedly inhibited at pH 8.5 (*p* < 0.0001). Total seedling length mirrored these patterns, with 7.0 showing the greatest overall growth and 8.5 the lowest (Fig. 9C). The hypocotyl-to-epicotyl ratio decreased sharply from 7 to 14 DAS (p < 0.0001; Table 3), indicating a developmental shift toward epicotyl investment over time (Fig. 9D). This ontogenic progression from hypocotyl elongation to epicotyl investment parallels the transition from heterotrophic to autotrophic growth, reflecting developmental shifts under contrasting substrate pH conditions. While the main pH effect on the ratio was not significant, the pH × DAS interaction was, with contrasting values at 7 DAS (5.5 > 7.0; 8.5 > 7.0; both *p* ≤ 0.012) that disappeared by 14 DAS. Panel D displays the log-transformed ratio for visualization, but statistical analyses were performed on raw data (Tables 3 and S14b).

**Table 3:** Effects of substrate pH, days after sowing (DAS), and their interaction on seedling growth traits.

Trait	Source	F	*p*-value	Significant contrasts (Tukey, *p* < 0.05)
Hypocotyl length	pH	65.02	< 0.0001	7.0 > 5.5; 5.5 > 8.5
	DAS	433.10	< 0.0001	14 > 7
Epicotyl length	pH	11.26	< 0.0001	8.5 > 5.5; 8.5 > 7.0
	DAS	2036.01	< 0.0001	14 > 7 (all pH)
	pH × DAS	12.09	< 0.0001	–
Total seedling length	pH	65.11	< 0.0001	7.0 > 5.5; 7.0 > 8.5
	DAS	1098.34	< 0.0001	14 > 7
Hypocotyl-to-epicotyl ratio	pH	1.90	0.1494	n.s
	DAS	618.09	< 0.0001	14 < 7 (all pH)
	pH × DAS	12.27	< 0.0001	–

Results of two-way ANOVA testing the effects of substrate pH, days after sowing (DAS; 7 vs. 14 days), and their interaction (pH × DAS) on seedling growth traits. F-values and p-values are shown for each source of variation. Significant pairwise contrasts (Tukey’s HSD, *p* < 0.05) for the main effects are indicated in the last column. Interaction terms are reported only when significant. All models met assumptions of homogeneity (Levene’s test, *p* > 0.05) and normality of residuals (Shapiro–Wilk test, *p* > 0.05).

Together, these results demonstrate that time after sowing primarily drives elongation, while substrate pH modulates organ-specific growth and allocation. Neutral pH (7.0) enhances hypocotyl and total elongation, whereas alkaline pH (8.5) constrains epicotyl development. Early differences in organ allocation tend to diminish as seedlings mature. These findings summarize the effects of substrate pH and time on early seedling development. Notably, alkaline stress at pH 8.5 mirrors the reduced early seedling establishment observed in MEC soil, linking laboratory and soil-based responses.

### Integrated effects of substrate pH, time after sowing, and dormancy group on early seedling establishment

Integration across dormancy, soil, and pH datasets enabled us to evaluate how multiple factors jointly shape early seedling establishment in *Lupinus*. Correlation analyses across experiments revealed that dormancy release, soil-based establishment metrics, and pH-related responses were largely independent (Spearman’s ρ =  − 0.19 to 0.13, all *q* > 0.10), indicating limited covariation among these components (Fig. S5). Accordingly, dormancy-related traits, soil-driven establishment, and pH responses represent largely independent axes of variation during early establishment, consistent with the operation of multiple, partially orthogonal filters. Dormancy group did not predict establishment outcomes across soil or pH conditions, supporting the use of a multivariate framework to integrate these components without assuming direct trait coupling.

**Fig. 10:**
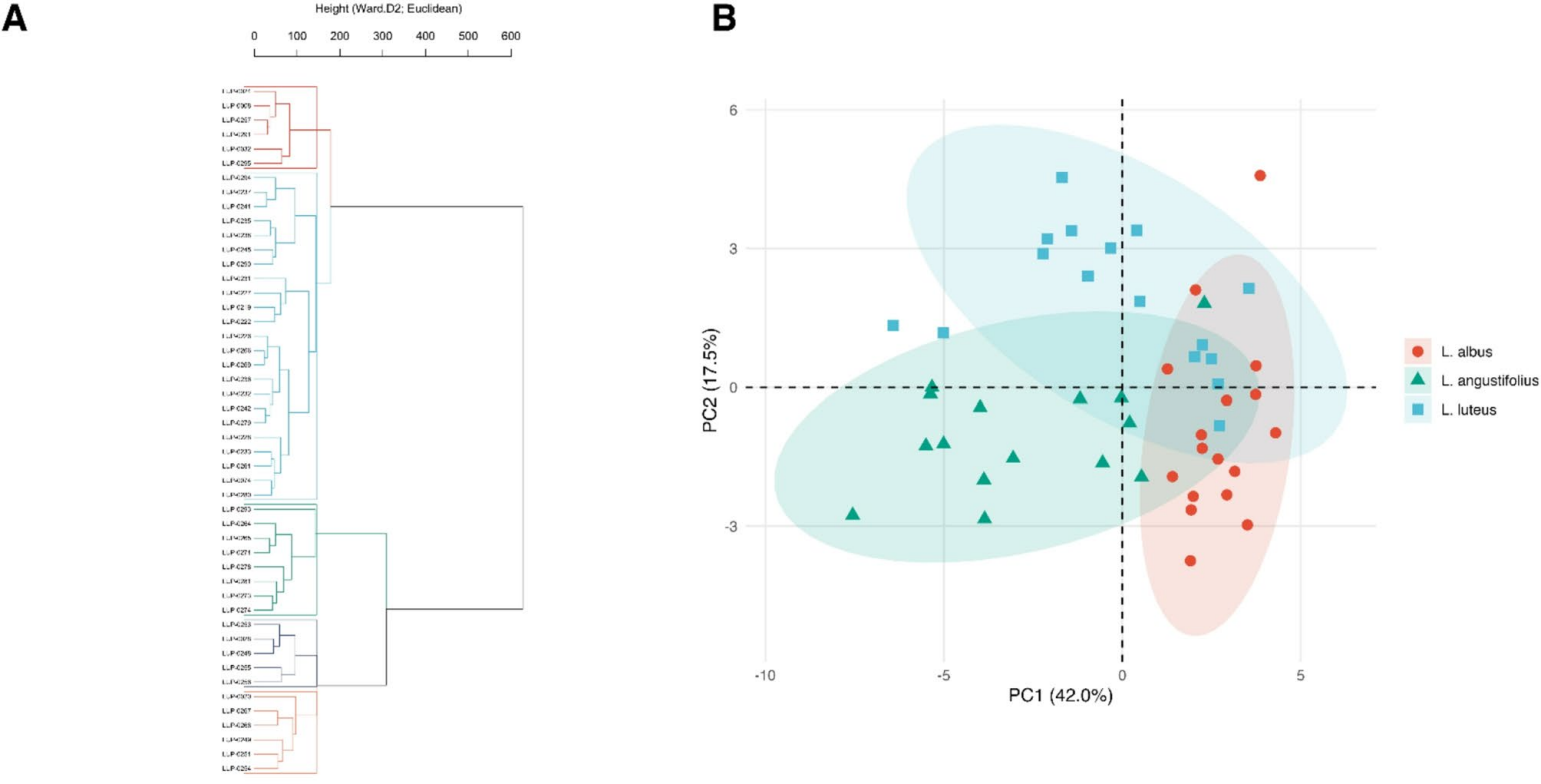
Multivariate phenotypic structure of *Lupinus* accessions based on germination, establishment, and growth traits. (**A**) Hierarchical clustering (Ward.D2) of 48 accessions based on standardized mean trait values of germination, early seedling establishment, and seedling growth measured across all experiments. Five major clusters (C1–C5) are color-coded; species identity is indicated. Branch colors denote cluster membership. Cluster composition is summarized in Table S15, including species identity and domestication status. (**B**) Principal component analysis (PCA) showing the distribution of accessions along the first two principal components (PC1 = 42.0%, PC2 = 17.5%). Shapes and colors denote species identity; ellipses represent 95% confidence regions per each species. All traits were z-score standardized prior to clustering and PCA. Accessions with extreme scores are labelled. See Table S16 for PCA loadings and trait contributions (panel B), Table S17 for accession PC scores, and Table S18 for per-accession trait summaries and the Integrated Selection Index used for comparative synthesis.

Hierarchical clustering of standardized phenotypic traits (germination, establishment, seedling growth) grouped the 48 accessions into five major clusters (Fig. 10A; Table S15). Cluster 2, composed mainly of *L. albus* landraces and cultivars with a few *L. luteus* accessions, represented the highest establishment and growth performance group across the measured traits, with predominantly positive PC1 scores. Cluster 4 consisted almost exclusively of wild *L. angustifolius* accessions with negative PC1 values and comparatively low germination and establishment success. Intermediate clusters were mixed in species composition, revealing partially convergent phenotypic response profiles across early establishment traits rather than strictly taxonomic segregation. Cluster 3, primarily *L. luteus* landraces and wild accessions, spanned both principal components, suggesting broader variation in early establishment and growth responses. The silhouette index (mean = 0.32) indicated moderate cluster structure. Species–cluster concordance was high for *L. albus* (100%) and *L. luteus* (93.8%) (Table S15).

Principal component analysis (PCA) on z-standardized traits retained the first two components based on the scree plot and parallel analysis (Fig. 10B; Fig. S6): PC1 = 42.0%, PC2 = 17.5% (cumulative 59.5%). PC1 captured a general gradient of germination and establishment performance across soil and substrate pH conditions, separating wild *L. angustifolius* accessions (PC1–) from *L. albus* accessions (PC1 +). PC2 reflected variation in seedling elongation dynamics and hypocotyl-to-epicotyl allocation, partially distinguishing accessions according to domestication status, with domesticated forms tending towards positive PC2 values but with substantial overlap. Trait contributions (loadings) are detailed in Table S16, and accession scores in Table S17. PCA and clustering were congruent, identifying species and domestication status as major structuring factors of phenotypic variation during early seedling establishment, while still showing substantial overlap among accessions. Together, these results indicate that variation in early establishment traits is structured along multiple, partially independent phenotypic axes, rather than being driven by a single dominant factor.

The integrated selection index (ISI; Table S18) summarized establishment success, seedling growth, and performance stability, derived exclusively from the substrate pH experiment (Experiment 3) under acidic (pH 5.5) and neutral (pH 7.0) conditions. High-ranking accessions included landraces and wild material showing consistent establishment and growth across pH conditions. Several wild *L. angustifolius* (e.g., LUP-0248, LUP-0249, LUP-0257) and *L. albus* landraces (e.g., LUP-0238) reached upper ISI values (> 0.55), reflecting robust establishment performance under contrasting substrate pH levels. In contrast, some cultivars such as LUP-0294 (*L. albus*) and LUP-0295 (*L. luteus*) scored lower, reflecting lower overall index values relative to accessions with more stable establishment across pH conditions. The ISI provides a comparative ranking of accessions based on multivariate early-establishment performance under controlled pH conditions. Clusters 2 and 3 concentrated most high-ranking accessions, whereas Clusters 1, 4, and 5 contained predominantly wild *L. angustifolius* with lower overall multivariate scores.

Accession-level recommendations integrating soil performance (Experiment 2) and responses under substrate pH gradients (Experiment 3) are summarized in Table 4. *L. albus* landraces LUP-0236 and LUP-0237 were best suited to neutral to alkaline soils, whereas *L. luteus* accessions LUP-0074 and LUP-0280 performed best under acidic conditions. The cultivar LUP-0293 (*L. angustifolius*) emerged as a strong candidate for highly alkaline MEC-like soils.

**Table 4:** Recommended *Lupinus* accessions for each soil type based on establishment performance in soil environments and response under substrate pH.

Soil code	Key soil features	Recommended accessions
BV	Neutral pH 7.3; OM very high; fertile reference	LUP-0219, LUP-0236, LUP-0237, LUP-0233, LUP-0241
COPMO	Alkaline pH 8.2; OM medium; clay loam	LUP-0228, LUP-0236, LUP-0233, LUP-0242
COPSA	Alkaline pH 8.3; OM low-medium; clay loam	LUP-0236, LUP-0228, LUP-0233, LUP-0290
MEC	Very alkaline pH 8.4; OM very low; loam; stress filter	LUP-0237, LUP-0290, LUP-0293, LUP-0226, LUP-0233
MBG3	Acidic pH 6.3; OM high; clay loam; humid climate	LUP-0232, LUP-0074, LUP-0280, LUP-0279

Key soil features include pH, organic matter (OM) content, and texture. Recommended accessions were identified based on (i) comparative establishment performance in the soil experiment (Experiment 2) and (ii) consistent establishment and growth responses under acidic and neutral substrate pH conditions (Experiment 3). Accessions showing high performance in both experiments were prioritized; when fewer than three overlaps occurred, the list was completed using the next best-performing accessions within each soil type. Soil classification followed Table 1.

Broader patterns from clustering and ISI (Table S18) were consistent with soil-dependent structuring of early establishment. Acidic, organic-rich soils (MBG3-like) favored *L. luteus* and several *L. albus* accessions, whereas neutral fertile soils (BV-like) supported a wider range of accessions across species. Alkaline soils with medium OM (COPMO/COPSA-like) showed consistent establishment and germination performance, while highly alkaline, low-OM soils (MEC-like) were the most restrictive and favored only a small subset of accessions with stable performance across environments (e.g., LUP-0290, LUP-0293, LUP-0237). Dormancy group was not associated with soil-specific performance patterns (Table S18).

Overall, early establishment in *Lupinus* was structured mainly by species identity and domestication status, with soil physicochemical properties modulating their phenotypic expression. Organic-rich substrates tended to buffer trade-offs among early establishment traits, whereas highly alkaline, low-OM soils imposed the strongest constraints. These patterns indicate that soil conditions act as major filters of early establishment under the experimental environments evaluated here.

## Discussion

Early establishment in *Lupinus* depended on the interaction between intrinsic seed traits and substrate conditions. By integrating standardized dormancy assays, multi-soil trials, and pH-gradient experiments, we show that seed dormancy, soil physicochemical properties, and substrate pH influence germination and early seedling development in different but complementary ways. This is particularly relevant under Mediterranean conditions, where strong seasonal contrasts in temperature, rainfall, and soil chemistry constrain the window for successful establishment.

### Physical dormancy as a determinant of early establishment variability

In *Lupinus angustifolius*, physical dormancy (PD) due to seed-coat impermeability varied widely among the sixteen accessions tested (Fig. 2; Table S4). Under non-scarified conditions, final germination ranged from almost complete to below 20%, confirming PD as a quantitative accession-dependent trait. Mechanical scarification markedly accelerated and synchronized germination across all accessions, indicating that dormancy was largely physical and reversible by disrupting the seed coat. These patterns are consistent with findings in other legumes such as *Phaseolus vulgaris* and *Medicago truncatula*, where variation in seed-coat permeability and structure underlies differences in physical dormancy^3,7,8^. Heritability estimates for PD in related legumes suggest moderate genetic control (h^2^ ≈ 0.4–0.6)^14^, indicating that selection for dormancy magnitude is feasible in breeding programs. In agronomic contexts, physical dormancy has increasingly been recognized as a functional trait rather than a breeding constraint, particularly in systems where delayed or staggered emergence can enhance persistence and re-establishment^29,30^. In *Lupinus*, the quantitative variation detected here likely reflects polygenic control of seed-coat traits, including testa thickness, palisade cell organization, and hilum-related structures, as reported for other *Fabaceae*. The broad variation observed—from strongly dormant wild accessions to nearly non-dormant cultivated forms—is consistent with domestication-associated shifts in dormancy levels, while retaining substantial within-species diversity. Rather than implying local adaptation per se, these results indicate that PD represents a flexible trait with clear agronomic relevance, whose expression can be shaped by genetic background and management context.

The pronounced response to scarification in accessions LUP-0032, LUP-0256, contrasted with the spontaneous germination of LUP-0024, LUP-0251, revealing contrasting levels of physical dormancy with clear agronomic implications. Accessions with high physical dormancy (PD) spread risk by delaying germination until favorable conditions occur, whereas accessions with low PD germinate rapidly and uniformly, a desirable profile for cover-crop establishment where pretreatments are impractical and synchrony is valuable. Principal-component analysis (Fig. S2; Table S7) confirmed that germination indices (MGT, IVG, DDS₅₀) form a continuous gradient of physical dormancy rather than discrete classes. This continuous distribution, together with the moderate heritability reported for PD in related legumes, is consistent with a polygenic architecture, involving largely additive effects and potentially minor epistatic contributions. Comparable patterns in other legumes indicate that PD variation mediates trade-offs between delayed emergence and rapid establishment, supporting its relevance for agronomic management without implying local adaptation^14,31^.

Because PD expression can be modulated by temperature and moisture, premature coat softening under warming/drying climates could trigger early germination followed by seedling mortality^6,32^. Breeding should therefore consider not only the magnitude of PD but also its environmental sensitivity, particularly under Mediterranean conditions characterized by strong seasonal variability. This phenotypic plasticity in dormancy expression, genetically grounded in structural variability of the seed-coat and permeability traits, could be exploited to develop accessions combining predictable germination with controlled responsiveness to external cues. Classifying accessions into low-, intermediate-, and high-dormancy groups based on MGT, IVG, and DDS₅₀ (Fig. 3; Table S5) provides a quantitative framework for selecting ideotypes that balance predictable germination with robust early establishment under variable conditions. Low-PD accessions are particularly valuable for direct seeding and low-input systems, where uniform emergence underpins cover-crop functions such as soil structure improvement, organic matter inputs, support of soil biological activity^1,17,19,33^. The marked intraspecific variation observed in *L. angustifolius* provides a foundation for breeding programs aiming to balance rapid emergence with controlled responsiveness to environmental cues under Mediterranean climates.

### Edaphic properties outweigh dormancy in shaping early establishment

When seeds were transferred from standardized assays to contrasting soils, dormancy level alone did not predict emergence or early establishment. This experiment therefore represents an early-stage expression of genotype × environment (G × E) interactions, where soil properties act as dominant external filters modulating inherent seed traits. Across five soils differing in texture, pH, and organic-matter content, establishment success depended primarily on soil physicochemical properties and species identity (Fig. 4; Table 1). Despite high nutrient availability and OM content, BV soil exhibited the lowest establishment probabilities, likely because of osmotic constraints associated with elevated electrical conductivity and exchangeable K rather than nutrient limitation. In contrast, MBG3 soil (acidic, high OM) and COPMO soil (alkaline, medium-OM) supported the highest establishment success, while MEC and COPSA (alkaline, low-OM) imposed the strongest edaphic constraints on early seedling development.

Heatmap and PCA analyses (Figs. 5 and 6) revealed species-specific patterns of soil-dependent establishment performance. *L. albus* and *L. luteus* consistently grouped as high performers in MBG3, COPMO, and COPSA, whereas most *L. angustifolius* accessions formed low-establishment clusters, with only a few exceeding 80% establishment success in MEC. These outcomes mirror long-established agronomic patterns, where *L. albus* performs best in fertile, slightly acidic soils, while *L. luteus* excels under acidic, sandy, and low-fertility conditions^15,29,34^. The close agreement between these classical ecological niches and our multi-soil results supports the relevance of edaphic compatibility for early establishment, integrating responses of dormancy and seedling vigor. Rather than implying local adaptation, these patterns indicate that the expression of early establishment traits depends on soil physicochemical context, underscoring the importance of evaluating accessions across contrasting environments.

Biotic interactions may further modulate these soil-driven responses, although they were not directly evaluated in this study. Legume–microbe associations are known to influence germination and early seedling vigor^35,36^, and the orchard soils used here—each with distinct management legacies—are likely to differ in microbial composition. Such variation could contribute to the contrasting establishment performance observed across sites. Correlation analyses confirmed that DDS₅₀ measured under standardized assays was a poor predictor of establishment success in soil environments (Fig. 7; Table S13), emphasizing that emergence under realistic conditions is a composite phenotype shaped by temperature fluctuations, soil water potential, and biotic interactions, in addition to intrinsic seed traits^6,37^. Such context-dependent expression of dormancy-related traits may contribute to risk spreading under Mediterranean climates characterized by irregular rainfall ^7,14^, without implying adaptive optimization.

Accordingly, selection for cover crops should move beyond the pursuit of uniformly low PD alone, toward identifying soil-compatible accessions whose trait combinations support both rapid emergence and tolerance to edaphic constraints. This approach aligns with the objectives of Mediterranean orchard management, where inter-row legumes are established to mitigate erosion, enhance infiltration, and sustain nutrient cycling^36,38^. Only accessions capable of germinating and establishing under local soil conditions can consistently deliver these ecosystem services.

The multi-soil approach adopted here bridges variation in seed traits with establishment performance under realistic substrates, offering a translational framework for selecting species and accessions suited to the heterogeneity of Mediterranean orchard soils. By explicitly linking G × E interactions to measurable establishment outcomes, our findings clarify why seed-trait assays alone cannot reliably predict soil performance and highlight the value of integrating physiological and edaphic information when identifying robust cover-crop material.

### Species-specific pH tolerance and developmental allocation

The pH-gradient experiment (Figs. 8 and 9; Fig. S4) showed that substrate pH acts as a major environmental filter during germination and early growth. Species responses were clearly differentiated: *L. luteus* maintained establishment success under acidic conditions, *L. albus* reached maximum seedling growth and establishment performance near neutral pH, and *L. angustifolius* showed reduced establishment success at both extremes, indicating a narrower response range under the tested pH conditions^31,33^. These patterns are consistent with well-established agronomic differences among species, and highlight that matching species to the prevailing pH of orchard soils is important for successful establishment.

Growth analyses revealed that developmental time was the dominant driver of elongation, while pH modulated organ-specific responses. Hypocotyl and total seedling length increased markedly from 7 to 14 DAS, whereas epicotyl elongation was strongly inhibited at pH 8.5 (Fig. 9B–C). The hypocotyl-to-epicotyl ratio decreased with time, reflecting the developmental shift from emergence to early leaf expansion. Its transient sensitivity to pH (Fig. 9D; Table 3) suggests physiological adjustment to nutrient and ion availability, including phosphorus availability and base cation balance^39^. These responses indicate context-dependent allocation during early growth, which may contribute to establishment under heterogeneous soil chemistry without implying broader ecological adaptation.

Importantly, tolerance to pH stress did not always coincide with high establishment performance in soil environments. Some accessions tolerated both acidic and alkaline substrate conditions yet performed poorly in specific soils, likely due to additional constraints such as salinity/ionic strength or biotic interactions. This decoupling supports the need for independent evaluation of dormancy, soil establishment performance, and pH response. The weak correlation among these axes also suggests that distinct physiological processes underlie dormancy release, ionic homeostasis, and early vigor—each potentially selectable but only partially overlapping.

Within *L. angustifolius*, variation in DDS₅₀ among accessions defines intrinsic thresholds that may interact with soil conditions and temperature to shape germination timing. More broadly, published studies in Lupinus demonstrate that performance can vary among populations in response to environmental change^40,41^; however, our study quantifies early establishment responses under controlled substrate pH and soil conditions, rather than testing evolutionary adaptation directly.

Under increasing climate variability, these species differences in pH response are agronomically relevant. Our results suggest that *L. luteus* is best suited for acidic soils where rapid emergence supports early cover; *L. albus* performs optimally in neutral substrates, combining high seedling growth and establishment performance; and selected *L. angustifolius* accessions with intermediate responses can be considered for soils where pH is less extreme and moisture conditions are favorable. Together, these findings show that substrate pH responses and developmental allocation patterns contribute to early establishment differences among Mediterranean *Lupinus* species, supporting context-specific species matching for orchard ground cover.

### An integrated framework for selecting resilient cover crops

By integrating results from all experiments, we developed an Integrated Selection Index (ISI) derived exclusively from the substrate pH experiment (Experiment 3) that combines early establishment success, seedling growth, and performance stability across acidic-to-neutral conditions (Fig. 10; Tables S15–S18). Calculated at DAS₁₄, the ISI integrates three components—mean establishment success at pH 5.5 and 7.0, total seedling length, and a stability score—to identify accessions with balanced performance under contrasting pH conditions. Importantly, physical dormancy and soil responses were not included in the index itself but were evaluated independently and interpreted alongside ISI rankings. High-index accessions combined vigorous early growth and consistent establishment across pH conditions, confirming that successful establishment in orchard substrates depends on composite performance rather than on any single trait. Because these component traits show moderate heritability in related legumes, the ISI provides a phenotypic descriptor of multivariate performance suitable for early-stage selection, rather than a direct measure of adaptation. The weak correlation between intrinsic dormancy metrics (e.g., DDS₅₀) and establishment under complex soil or pH conditions (Fig. S5) underscores the need to assess multiple environmental filters—chemical, physical, and biotic—alongside seed traits, rather than relying on dormancy metrics alone.

Cluster analysis provided an additional layer of interpretability: Clusters 2 and 3, mainly comprising *L. albus* landraces/cultivars and *L. luteus* accessions, contained most high-performing accessions, whereas Clusters 1, 4, and 5 (dominated by wild *L. angustifolius*) were associated with lower multivariate performance (Fig. 10; Table S15). This pattern indicates that species identity and domestication status are major axes structuring early establishment phenotypes, while still allowing substantial overlap among groups. The congruence between PCA and clustering suggests that phenotypic variation across environments, rather than single diagnostic traits, underlies the observed performance gradients.

These findings establish a quantitative, phenotype-based framework for legume cover cropping in Mediterranean citrus orchards. Previous work shows that cover crops improve soil structure, infiltration, and nutrient cycling^17,18,42^, while legumes contribute nitrogen inputs and support soil biological activity^19^. By linking early establishment traits measured under controlled pH conditions with performance observed in contrasting soils, the ISI facilitates the strategic deployment of lupins—acid-tolerant *L. luteus*, high-biomass *L. albus*, and selected *L. angustifolius* accessions—across heterogeneous orchard substrates. Because the ISI integrates only partially correlated traits, it provides a practical descriptor of resilience at the establishment stage.

Our multi-species, multi-environment design represents, to our knowledge, the first integrative framework tailored to lupin deployment in perennial systems. The practical implications are direct: species and accessions can be allocated to orchard blocks according to their composite early-establishment profiles. For example, *L. luteus* is suited to acidic substrates where rapid emergence is essential; *L. albus* performs best in neutral to slightly acidic substrates, producing vigorous seedlings and high biomass^24,43^; and selected *L. angustifolius* accessions combining low dormancy and moderate pH tolerance establish well in moderately alkaline soils under adequate moisture. This trait-to-site matching illustrates predictive phenotyping at the establishment stage, bridging controlled assays with agronomic deployment.

As climate change alters the thermal and moisture cues governing dormancy release and germination^6,32^, composite ideotypes should be prioritized over single-trait targets. The ISI framework supports this shift by emphasizing integrated early performance rather than presumed adaptation. Several challenges remain. Multi-year trials are required to assess seasonal stability, and explicit characterization of soil microbiota could clarify soil–genotype interactions^44,45^. Although our assays encompassed multiple soil types, they were conducted within a single season and under controlled conditions. Extending these evaluations across years and locations will be essential to capture dormancy carryover, soil legacy effects, and establishment robustness under real-world management. In addition, the soil microbiome, uncharacterized here, likely contributes to differential establishment success; its integration would enable explicit testing of genotype × soil × microbiota interactions and refine site-specific recommendations^35,36^. Physical dormancy levels can be influenced by the maternal environment during seed development, introducing environmentally driven variation among seed lots that was not explicitly evaluated in our design^46^. Finally, anatomical and genomic analyses—seed-coat structure, permeability traits, and candidate loci from GWAS or transcriptomics—could clarify the genetic architecture underlying dormancy variation, pH responses, and early growth^15,46, 47]^, helping to integrate phenotypic indices such as ISI with marker-assisted selection strategies.

Finally, validating this approach across Mediterranean orchard systems—olive, almond, and vineyard—will test its broader applicability. All face similar constraints, including erosion risk, low organic matter, and the need for compatible legume covers^17,19^. By explicitly separating dormancy, soil performance, and pH response while interpreting them jointly, the ISI framework offers a replicable and conceptually transparent model for designing sustainable ground-cover strategies in perennial Mediterranean systems under increasing climatic uncertainty.

## Conclusion

By integrating dormancy profiling, soil-type responses, and pH tolerance within a unified experimental design, this study delivers the first cross-species phenotypic assessment of early establishment traits in *Lupinus* using citrus-derived and reference soils under controlled conditions. Across species and accessions, we identified material combining low physical dormancy, soil-compatible establishment, and stable performance across acidic-to-neutral pH conditions—attributes essential for reliable establishment in Mediterranean orchards. The Integrated Selection Index (ISI) introduced here is derived exclusively from the substrate pH experiment and provides a reproducible, trait-based metric of early establishment performance, while dormancy and soil responses are evaluated independently and interpreted jointly at a conceptual level.

These findings support a transition from empirical variety testing to predictive phenotyping approaches. Because the ISI combines partially independent components of early establishment, it serves as a synthetic descriptor of multivariate performance suitable for early-stage selection, without implying evolutionary adaptation. Beyond citrus, the framework is scalable to other perennial systems—olive groves, vineyards, and almond orchards—that face similar challenges in cover establishment, nitrogen inputs, and erosion control. By explicitly separating dormancy, soil performance, and pH response while interpreting them together, this work provides a transparent basis for selecting *Lupinus* species and accessions suited to heterogeneous Mediterranean soils. Together, these results position *Lupinus* as a useful system for linking phenotypic plasticity with early establishment outcomes under variable soil conditions. Future work integrating multi-year field trials and soil biotic characterization will be essential to extend these conclusions beyond controlled conditions and to refine site-specific recommendations under increasing climatic uncertainty.

## Supplementary Information

Below is the link to the electronic supplementary material.

Supplementary Material 1

## Data Availability

The datasets generated and analyzed during the current study are available from the corresponding author upon reasonable request.
