# Supplementary material for: Genetic variation in seed dormancy, soil tolerance, and pH response jointly shape early establishment in Lupinus species
Source: Sci Rep. 2026 Apr 1;16:15317. doi: 10.1038/s41598-026-46460-7 (PMC13181099; doi:10.1038/s41598-026-46460-7)
Supplement: Supplementary file 1 — Supplementary Material 1 [file 41598_2026_46460_MOESM1_ESM.docx]

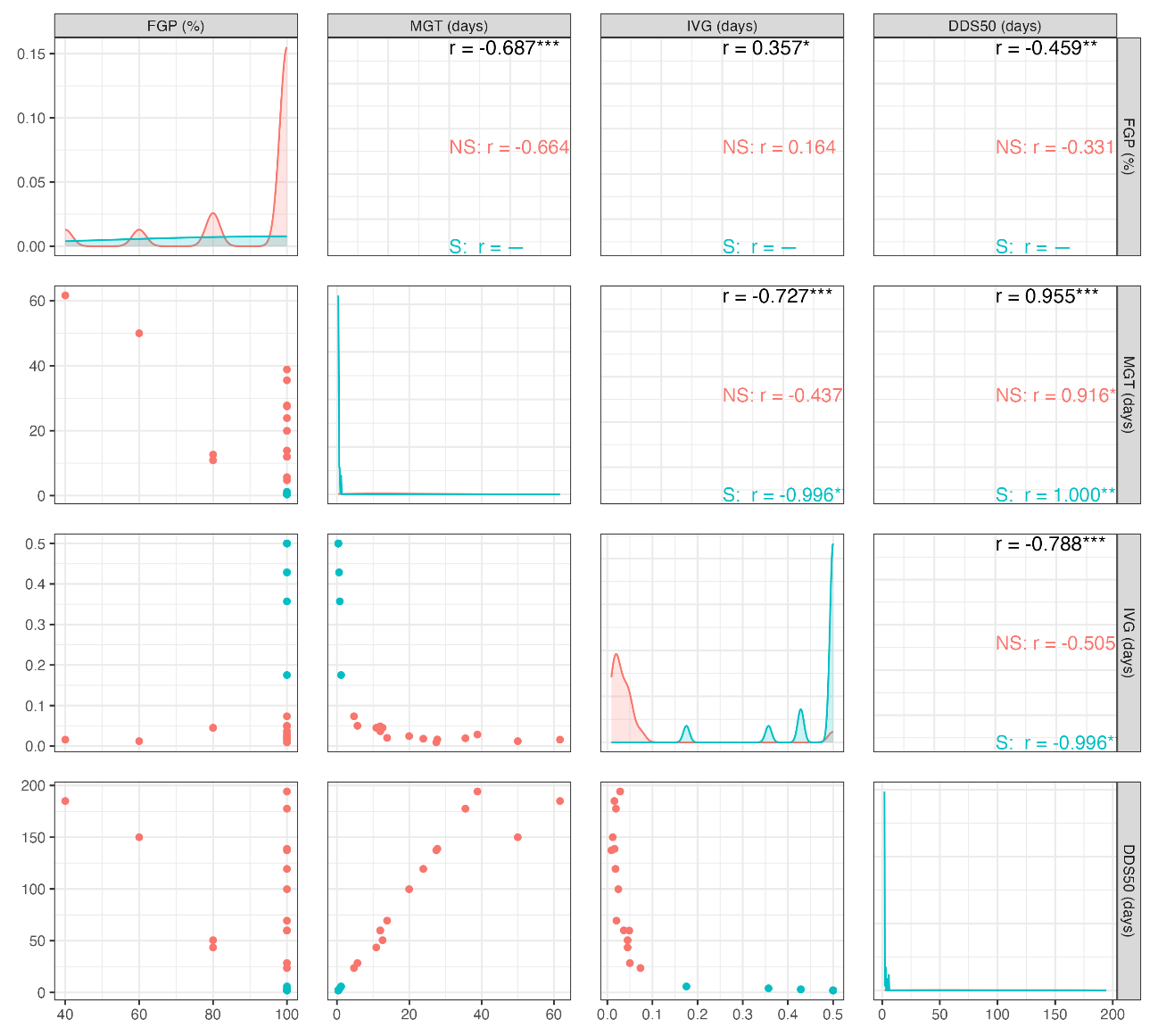


**Figure S1. Pearson correlation matrix among four germination parameters (FGP, MGT, IVG, and DDS₅₀) in *L. angustifolius* accessions.** The diagonal shows histograms of each variable. The upper triangle displays overall Pearson correlation coefficients (r) calculated across accessions, with significance levels (*** *p <* 0.001, ** *p <* 0.01, * *p <* 0.05, ns = not significant); the lower triangle shows scatter plots by treatment (non-scarified, NS = red; scarified, S = blue) to visualize treatment-specific patterns. A dash (–) indicates correlations not computed due to lack of variation. See Table S6 for corresponding Pearson coefficients.


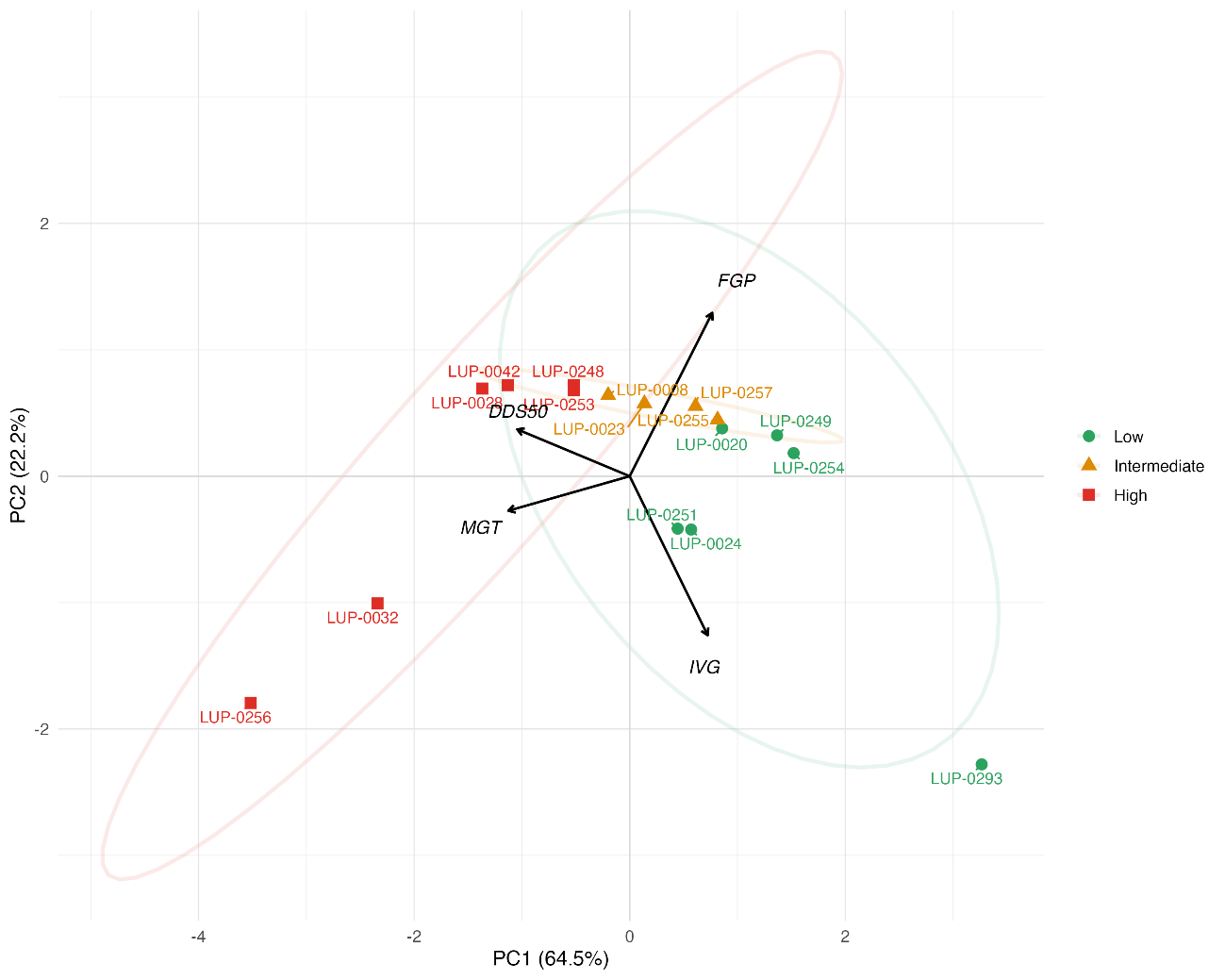


**Figure S2. Principal component analysis (PCA) of germination traits of 16 *L. angustifolius* accessions under non-scarified (NS) conditions.** Variables include MGT (mean germination time), IVG (germination velocity index), DDS₅₀ (days to 50 % germination), and FGP (final germination percentage). Points are labelled by accession and colored according to dormancy group (Low, Intermediate, High) defined from multivariate patterns under NS conditions. Ellipses indicate 95 % confidence regions of accession dispersion. Black arrows represent variable loadings on the PC1–PC2 plane, showing each trait’s contribution and direction. See Table S7 for PCA variance and loadings.


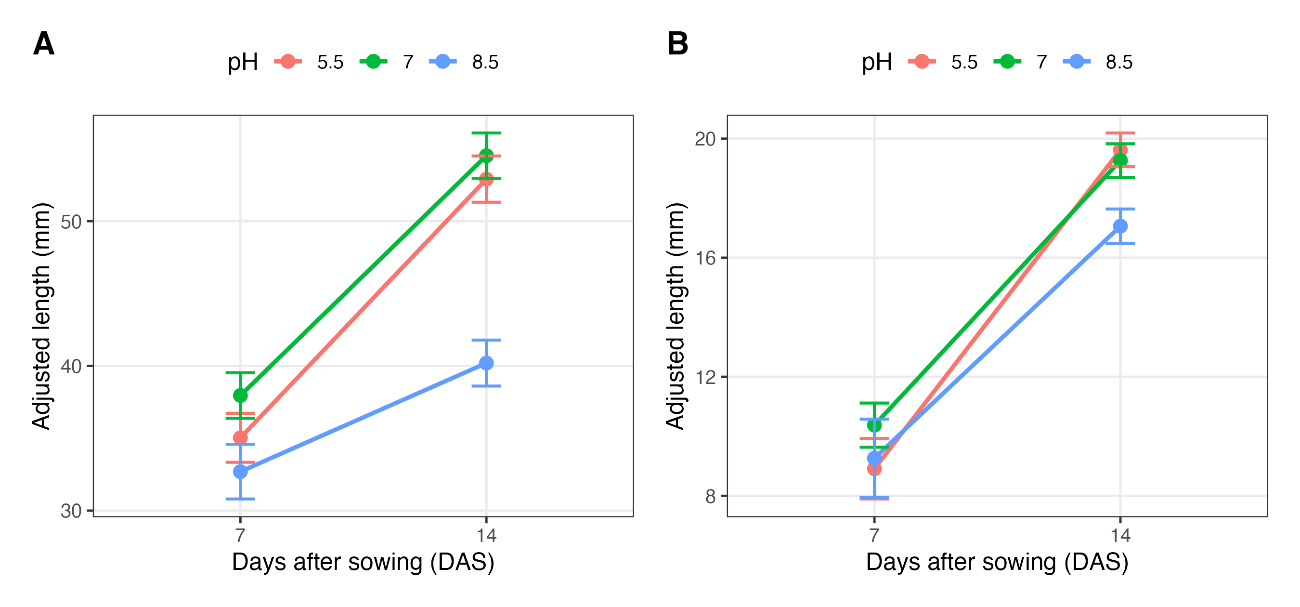


**Fig. S3**. **Interaction effects of substrate pH and time after sowing on growth traits.** (**A**) Hypocotyl length and (**B**) epicotyl length. Each line connects the mean trait value at 7 and 14 days after sowing (DAS) for each pH level (5.5, 7.0, 8.5). Points represent least-squares means (± 95% CI) estimated from two-way ANOVA models with pH and DAS as fixed factors. The pH × DAS interaction was not significant for hypocotyl length (*F* = 0.36, *p* = 0.547), whereas it was significant for epicotyl length (*F* = 11.98, *p* = 0.0005).


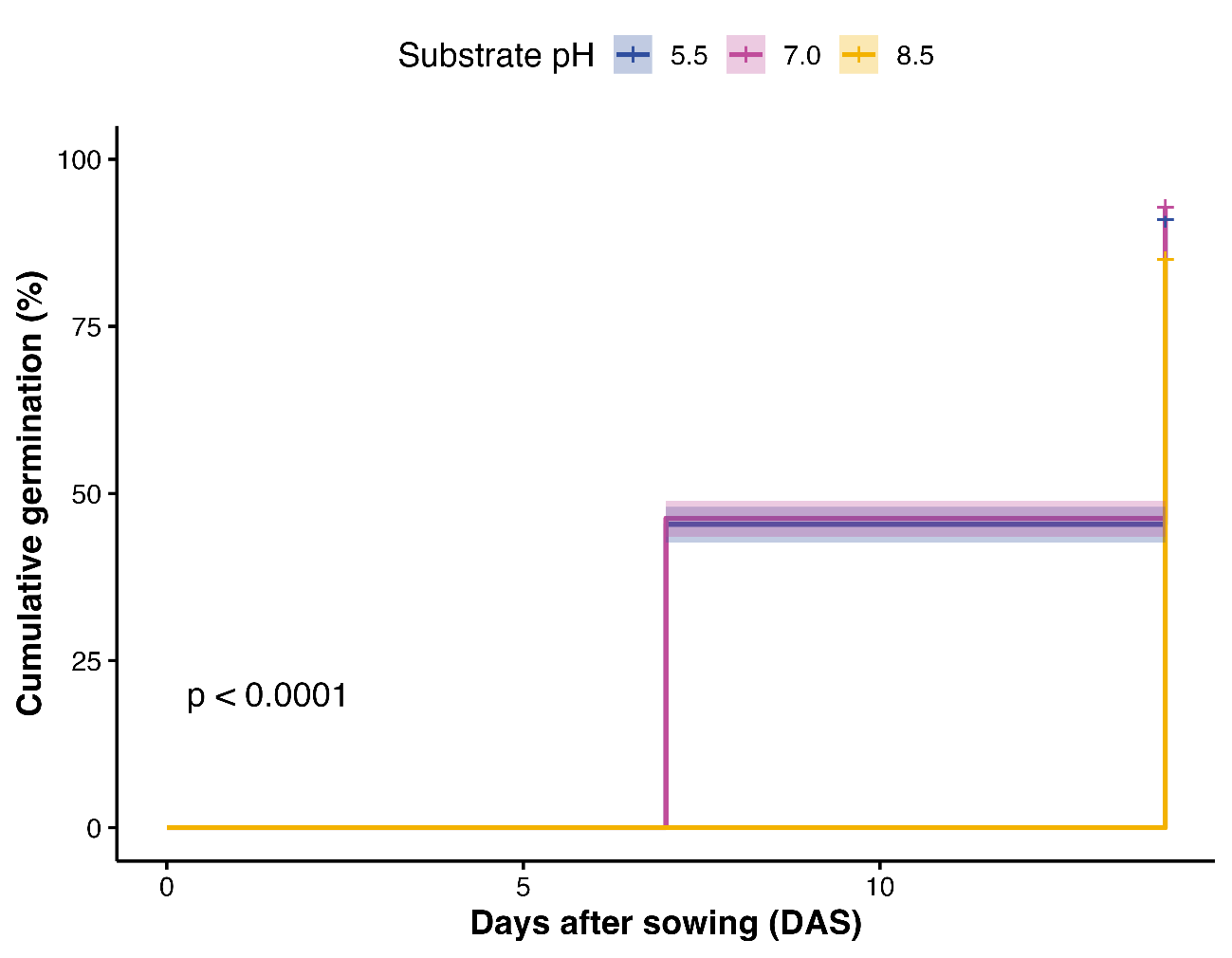


**Fig. S4**. **Cumulative germination curves under different substrate pH levels (5.5, 7.0, 8.5) pooled across the three Lupinus species.** Kaplan–Meier curves show the cumulative proportion of germinated seeds (%) over time (days after sowing, DAS). Shaded areas represent 95 % confidence intervals, and tick marks indicate censored seeds. A log-rank test indicated significant differences among pH levels (χ² = 354, df = 2, *p <* 0.0001), indicating delayed germination under alkaline conditions (pH 8.5) relative to acidic and near-neutral substrates.


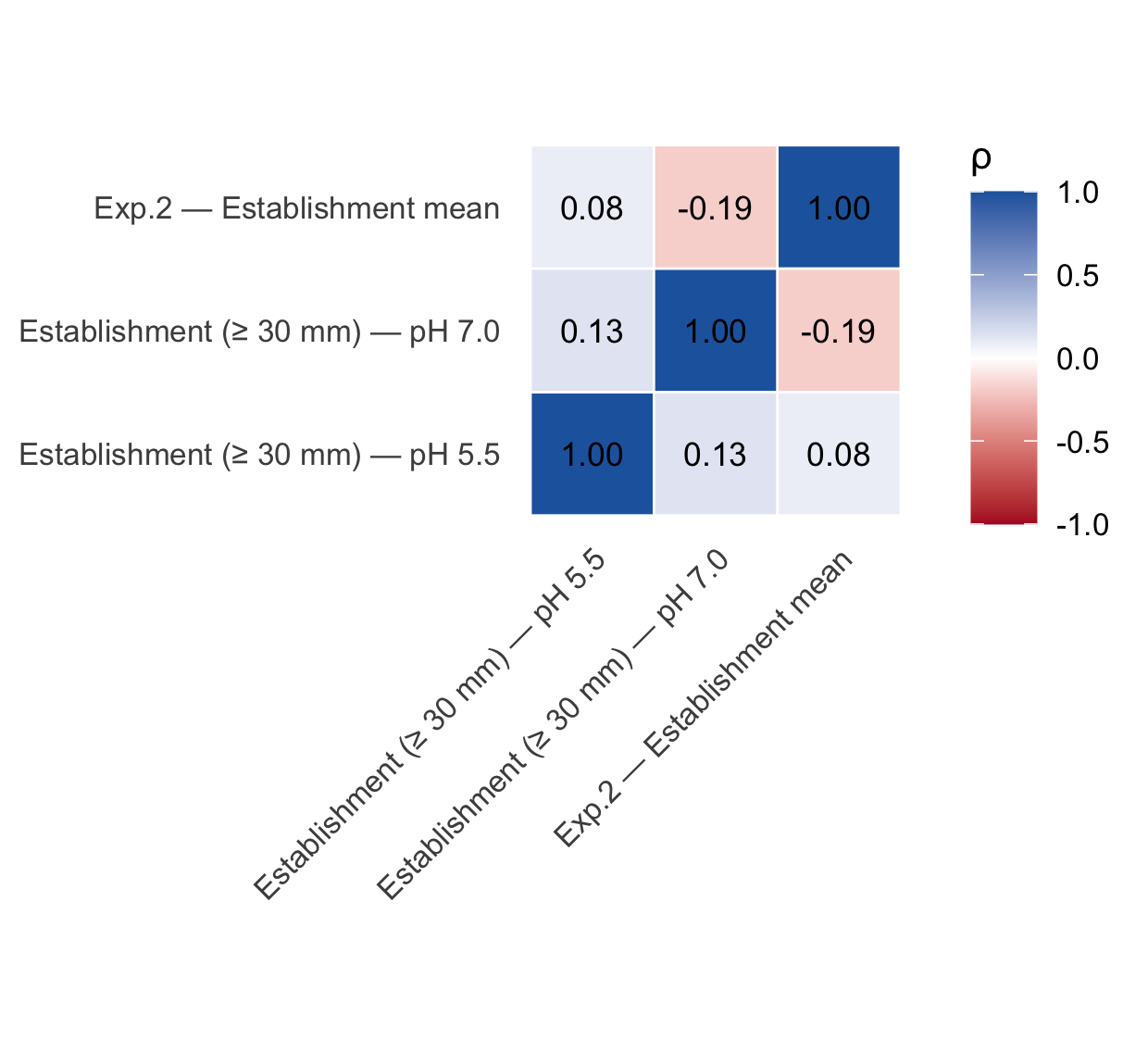


**Fig. S5. Spearman correlation heatmap of early seedling establishment metrics across 48 *Lupinus* accessions.** Pairwise correlations were computed between mean early seedling establishment across the five soil types (Experiment 2) and establishment success under acidic (pH 5.5) and neutral (pH 7.0) conditions (Experiment 3). Cells show Spearman’s rank correlation coefficients (ρ), with the color scale indicating magnitude and sign. *P*-values were adjusted for multiple testing using the false discovery rate (FDR); no correlations remained significant after FDR correction.


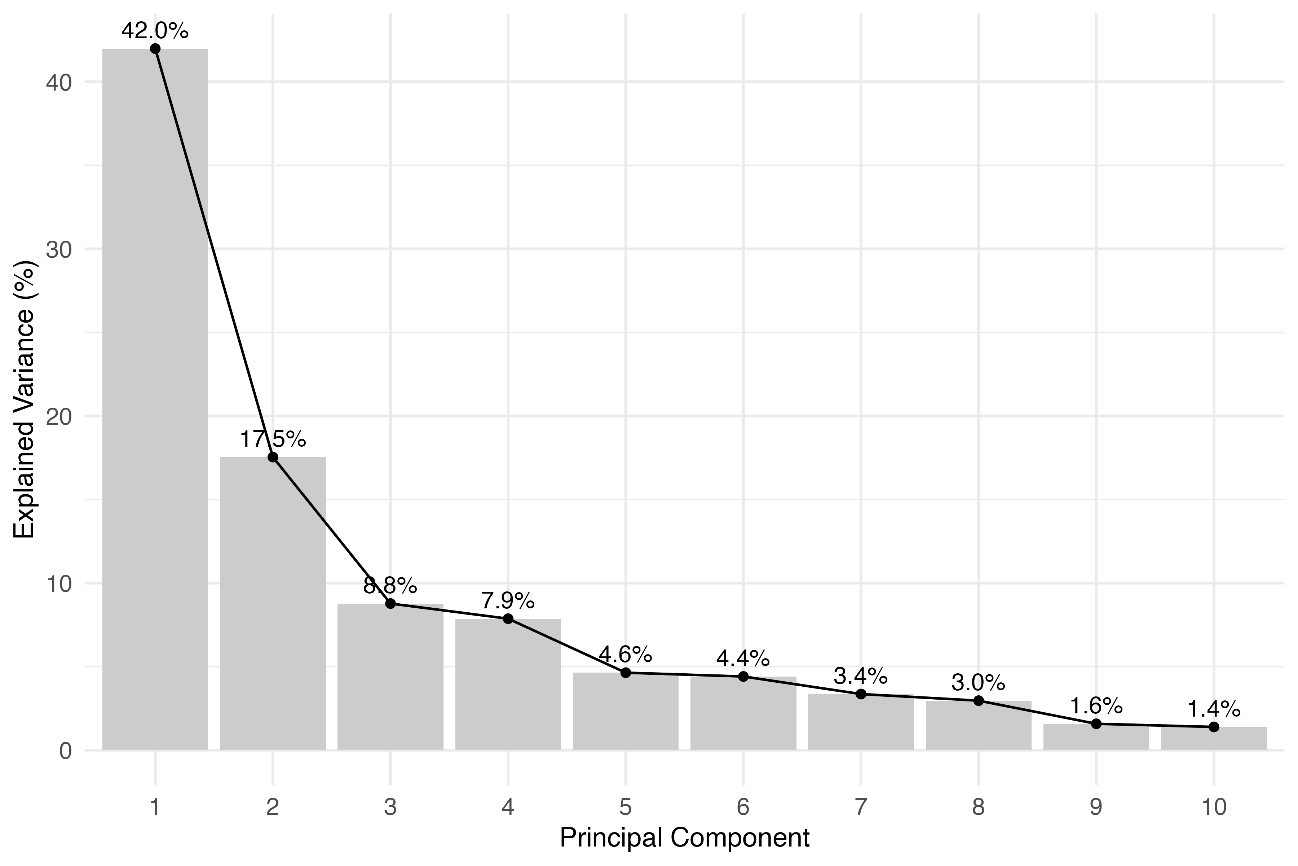


**Fig. S6. Scree plot of the principal component analysis (PCA) for 48 *Lupinus* accessions**. Eigenvalues for the first ten principal components are shown. PC1 and PC2 explained 42.0 % and 17.5 % of the total variance, respectively, and were retained for visualization in Figure 10B (cumulative variance = 59.5 %).

**Table S1.** Passport information for the 50 *Lupinus* accessions used in the study, including geographic origin, coordinates, altitude, biological status (wild, landrace, or cultivar), species, gene bank identifiers, 100-seed weight, and participation in each experiment.

| **Accession** | **Geographic origin** | **Coordinates** | **Altitude (m)** | **Biological status** | **Species** | **Code CICYTEX (LO)** | **Code CRF (BGE)** | **Code NUMCAT (NC)** | **100-seed weight (g)** | **Experiments** |
| --- | --- | --- | --- | --- | --- | --- | --- | --- | --- | --- |
| LUP-0219 | Badajoz (Spain) | 38.153954°N, 6.213596°W | 630 | Landrace | *L. albus* | LO 03969 | BGE001672 | NC008311 | 38.0 | 2, 3 |
| LUP-0222 | Salamanca (Spain) | 40.935805°N, 6.717478°W | 687 | Landrace | *L. albus* | LO 03984 | BGE037416 | NC071066 | 46.0 | 2, 3 |
| LUP-0226 | Palencia (Spain) | 42.340825°N, 4.278199°W | 789 | Landrace | *L. albus* | LO 04168 | BGE004180 | NC052727 | 29.9 | 2, 3 |
| LUP-0227 | Zamora (Spain) | 41.616328°N, 6.088441°W | 759 | Landrace | *L. albus* | LO 04171 | BGE004281 | NC069482 | 35.5 | 2, 3 |
| LUP-0228 | Salamanca (Spain) | 40.608552°N, 6.464638°W | 707 | Wild | *L. albus* | LO 04432 | BGE001130 | NC080572 | 33.4 | 2, 3 |
| LUP-0231 | Ciudad Real (Spain) | 38.725663°N, 4.970771°W | 360 | Landrace | *L. albus* | LO 04766 | BGE011764 | NC008328 | 37.7 | 2, 3 |
| LUP-0232 | Cáceres (Spain) | 39.824435°N, 6.138883°W | 409 | Landrace | *L. albus* | LO 04790 | BGE025302 | NC011118 | 47.0 | 2, 3 |
| LUP-0233 | Cáceres (Spain) | 39.527095°N, 5.631265°W | 571 | Landrace | *L. albus* | LO 05246 | BGE029750 | NC011539 | 52.0 | 2, 3 |
| LUP-0235 | Valladolid (Spain) | 41.736694°N, 5.325465°W | 707 | Wild | *L. albus* | LO 06259 | BGE034346 | NC011865 | 40.5 | 2, 3 |
| LUP-0236 | Badajoz (Spain) | 38.619979°N, 6.797993°W | 329 | Landrace | *L. albus* | LO 06260 | BGE035464 | NC011871 | 38.0 | 2, 3 |
| LUP-0237 | Sevilla (Spain) | 37.327449°N, 5.416520°W | 134 | Landrace | *L. albus* | LO 06291 | BGE037811 | NC011880 | 54.2 | 2, 3 |
| LUP-0238 | León (Spain) | 42.239499°N, 5.375401°W | 832 | Landrace | *L. albus* | LO 09505 | BGE043860 | NC001691 | 35.5 | 2, 3 |
| LUP-0241 | Faro (Portugal) | 37.216217°N, 7.446124°W | 24 | Landrace | *L. albus* | LO 04101 | BGE002587 | NC004566 | 35.5 | 2, 3 |
| LUP-0242 | Évora (Portugal) | 38.801878°N, 7.456871°W | 427 | Landrace | *L. albus* | LO 04105 | BGE002599 | NC008880 | 43.6 | 2, 3 |
| LUP-0245 | Bragança (Portugal) | 41.537514°N, 6.508744°W | 567 | Landrace | *L. albus* | LO 04174 | BGE004935 | NC076357 | 35.0 | 2, 3 |
| LUP-0290 | Alicante (Spain) | 38.722979°N, 0.192514°W | 553 | Landrace | *L. albus* | LO 03904 | BGE038419 | NC071022 | 42.7 | 2, 3 |
| LUP-0294 | NA | NA | NA | Cultivar | *L. albus* | NA | NA | NA | 26.0 | 2, 3 |
| LUP-0008 | Pontevedra (Spain) | 42.236527°N, 8.088190°W | 416 | Wild | *L. angustifolius* | NA | BG002364 | NA | 5.4 | 1, 2, 3 |
| LUP-0020 | Ourense (Spain) | 42.347873°N, 7.256458°W | 755 | Wild | *L. angustifolius* | NA | BG002417 | NA | 9.5 | 1, 2, 3 |
| LUP-0023 | Pontevedra (Spain) | 41.905395°N, 8.858680°W | 44 | Wild | *L. angustifolius* | NA | BG002455 | NA | 6.1 | 1 |
| LUP-0024 | Ourense (Spain) | 42.267911°N, 8.152127°W | 104 | Wild | *L. angustifolius* | NA | BG002461 | NA | 8.7 | 1, 2, 3 |
| LUP-0028 | Lugo (Spain) | 42.846627°N, 6.900578°W | 1039 | Wild | *L. angustifolius* | NA | BG003174 | NA | 7.0 | 1, 2, 3 |
| LUP-0032 | A Coruña (Spain) | 42.861989°N, 8.540865°W | 238 | Wild | *L. angustifolius* | NA | BG003185 | NA | 6.2 | 1, 2, 3 |
| LUP-0042 | A Coruña (Spain) | 42.861989°N, 8.540865°W | 238 | Wild | *L. angustifolius* | NA | BG003537 | NA | 4.2 | 1 |
| LUP-0248 | Huelva (Spain) | 37.891495°N, 6.478384°W | 507 | Wild | *L. angustifolius* | LO 04930 | BGE001626 | NC004447 | 13.0 | 1, 2, 3 |
| LUP-0249 | Badajoz (Spain) | 38.268697°N, 6.474105°W | 497 | Wild | *L. angustifolius* | LO 04932 | BGE001636 | NC008263 | 11.8 | 1, 2, 3 |
| LUP-0251 | Salamanca (Spain) | 40.471077°N, 5.945591°W | 735 | Wild | *L. angustifolius* | LO 04997 | BGE001782 | NC009667 | 12.0 | 1, 2, 3 |
| LUP-0253 | Granada (Spain) | 36.994754°N, 3.079592°W | 959 | Landrace | *L. angustifolius* | LO 06229 | BGE025298 | NC011839 | 10.8 | 1, 2, 3 |
| LUP-0254 | Vila Real (Portugal) | 41.328046°N, 7.753020°W | 523 | Wild | *L. angustifolius* | LO 04974 | BGE005163 | NC008396 | 11.0 | 1, 2, 3 |
| LUP-0255 | Beja (Portugal) | 38.262851°N, 7.995576°W | 248 | Wild | *L. angustifolius* | LO 05068 | BGE003346 | NC011040 | 16.2 | 1, 2, 3 |
| LUP-0256 | Bragança (Portugal) | 41.518430°N, 6.279652°W | 672 | Wild | *L. angustifolius* | LO 05086 | BGE004823 | NC078799 | 13.0 | 1, 2, 3 |
| LUP-0257 | Évora (Portugal) | 38.720792°N, 7.983182°W | 319 | Wild | *L. angustifolius* | LO 05229 | BGE002971 | NC011528 | 17.5 | 1, 2, 3 |
| LUP-0291 | Cataluña (Spain) | 41.869486°N, 2.418046°E | 752 | Wild | *L. angustifolius* | LO 5276 | BGE005829 | NC010420 | 6.7 | 2, 3 |
| LUP-0293 | NA | NA | NA | Cultivar | *L. angustifolius* | NA | NA | NA | 17.3 | 1, 2, 3 |
| LUP-0074 | Ourense (Spain) | 42.347873°N, 7.256458°W | 755 | Wild | *L. luteus* | NA | BG002251 | NA | 10.6 | 2, 3 |
| LUP-0261 | Pontevedra (Spain) | 42.747510°N, 8.492981°W | 159 | Landrace | *L. luteus* | LO 04518 | BGE002220 | NC011436 | 9.7 | 2, 3 |
| LUP-0264 | Huelva (Spain) | 37.499704°N, 7.272342°W | 219 | Landrace | *L. luteus* | LO 04572 | BGE001621 | NC012397 | 17.9 | 2, 3 |
| LUP-0265 | Cáceres (Spain) | 40.221120°N, 6.878676°W | 476 | Landrace | *L. luteus* | LO 04576 | BGE001761 | NC012398 | 13.8 | 2, 3 |
| LUP-0266 | Cáceres (Spain) | 40.221120°N, 6.878676°W | 476 | Landrace | *L. luteus* | LO 04577 | BGE001762 | NC013856 | 9.3 | 2, 3 |
| LUP-0267 | Asturias (Spain) | 43.529988°N, 6.675090°W | 94 | Wild | *L. luteus* | LO 04659 | BGE002090 | NC013744 | 7.8 | 2, 3 |
| LUP-0268 | Lugo (Spain) | 43.126084°N, 7.068860°W | 952 | Landrace | *L. luteus* | LO 04751 | BGE002138 | NC008272 | 9.7 | 2, 3 |
| LUP-0269 | Huelva (Spain) | 37.495933°N, 7.269221°W | 219 | Landrace | *L. luteus* | LO 06290 | BGE036437 | NC011878 | 12.6 | 2, 3 |
| LUP-0271 | Faro (Portugal) | 37.324466°N, 7.636733°W | 164 | Landrace | *L. luteus* | LO 04583 | BGE002655 | NC013988 | 13.9 | 2, 3 |
| LUP-0273 | Portalegre (Portugal) | 39.123777°N, 7.288152°W | 272 | Landrace | *L. luteus* | LO 04591 | BGE002666 | NC008277 | 11.3 | 2, 3 |
| LUP-0274 | Santarém (Portugal) | 38.937119°N, 8.501032°W | 32 | Wild | *L. luteus* | LO 04605 | BGE002965 | NC008278 | 11.0 | 2, 3 |
| LUP-0278 | Beja (Portugal) | 38.205550°N, 7.920154°W | 183 | Wild | *L. luteus* | LO 04625 | BGE003005 | NC009754 | 12.8 | 2, 3 |
| LUP-0279 | Beja (Portugal) | 37.652817°N, 8.223976°W | 223 | Wild | *L. luteus* | LO 04627 | BGE003007 | NC012205 | 13.3 | 2, 3 |
| LUP-0280 | Faro (Portugal) | 37.316231°N, 8.831710°W | 54 | Landrace | *L. luteus* | LO 04664 | BGE002648 | NC011845 | 11.7 | 2, 3 |
| LUP-0281 | Évora (Portugal) | 38.425186°N, 7.537921°W | 220 | Landrace | *L. luteus* | LO 04695 | BGE002746 | NC012188 | 13.0 | 2, 3 |
| LUP-0295 | NA | NA | NA | Cultivar | *L. luteus* | NA | NA | NA | 14.5 | 2, 3 |

Coordinates are given in decimal degrees (latitude, longitude; N/S and E/W indicated). 100-seed weight values follow collection records or bulk measurements when available. NA indicates data not available. Biological status reflects the origin of each accession (wild, landrace, or cultivar) and is used in the interpretation of establishment responses, while accessions are analyzed as reference populations rather than genetically uniform lines. Experiment codes indicate the assays in which each accession was included: 1, physical dormancy (Experiment 1); 2, soil-based establishment (Experiment 2); and 3, pH-gradient establishment (Experiment 3). Gene bank identifiers include accession codes from the MBG-CSIC collection (LUP), the Centro de Investigaciones Científicas y Tecnológicas de Extremadura (CICYTEX; LO), and the Centro de Recursos Fitogenéticos (CRF, INIA-CSIC; BGE and National Inventory numbers [NUMCAT; NC]).

**Table S2.** Geographic origin and site-specific environmental characteristics of the five agricultural soils used in the soil-based establishment experiment.

| **Soil code** | **MEC** | **BV** | **COPSA** | **COPMO** | **MBG3** |
| --- | --- | --- | --- | --- | --- |
| **Province (Spain)** | Valencia | Tarragona | Alicante | Alicante | Pontevedra |
| **Location (lat., long.)** | 39.601531°N, 0.476654°W | 40.853599°N, 0.526214°E | 38.847100°N, 0.098900°W | 38.839935°N, 0.098931°W | 42.406900°N, 8.642700°W |
| **Altitude (m)** | 103 | 21 | 30 | 44 | 32 |
| **Precipitation (mm)** | 224 | 560 | 389 | 389 | 1083 |
| **Mean annual temperature (ºC)** | 19.0 | 17.5 | 19.1 | 19.1 | 14.6 |
| **Current crop** | Citrus orchards | Citrus orchards | Citrus orchards | Citrus orchards | Legume cropping system |

Geographic coordinates are provided in decimal degrees (latitude, longitude; N/S and E/W indicated). Current crop indicates the dominant land use at each sampling site. Soils were collected from five agricultural sites in Spain (MEC, BV, COPSA, COPMO and MBG3).

**Table S3.** Number (n) and percentage (%) of seedlings for which both hypocotyl and epicotyl lengths were recorded under different substrate pH conditions and incubation times.

| **DAS** | **pH 5.5** | |  | **pH 7.0** | |  | **pH 8.5** | |
| --- | --- | --- | --- | --- | --- | --- | --- | --- |
|  | **n** | **%** |  | **n** | **%** |  | **n** | **%** |
| DAS7 | 539 | 74.9 |  | 623 | 86.5 |  | 433 | 60.1 |
| DAS14 | 604 | 83.9 |  | 626 | 86.9 |  | 612 | 85.0 |

Number (n) and percentage (%) of seedlings showing complete hypocotyl and epicotyl development at pH 5.5, 7.0, and 8.5 after 7 (DAS7) and 14 (DAS14) days of incubation. The initial sample size per pH × DAS combination was 720 seeds. Seedlings lacking either measurement (n = 883) were excluded from growth and allocation analyses.

**Table S4**. Two-way ANOVA for germination parameters in 16 *L. angustifolius* accessions under scarified (S) and non-scarified (NS) conditions.

| **Source** | **Df** | **MGT** | | | | |  | **IVG** | | | | | |  | **DDS_50_** | | | |  |
| --- | --- | --- | --- | --- | --- | --- | --- | --- | --- | --- | --- | --- | --- | --- | --- | --- | --- | --- | --- |
|  |  | **SS** | **MS** | **F value** | ***p*-value** |  | **SS** | | **MS** | **F value** | ***p*-value** |  | **SS** | | | **MS** | **F value** | ***p*-value** |  |
| Accession | 15 | 5665.24 | 377.68 | 2.54 | ** |  | 0.98 | | 0.07 | 18.45 | *** |  | 116618.50 | | | 7774.57 | 3.07 | *** |  |
| Treatment | 1 | 15717.73 | 15717.73 | 105.52 | *** |  | 5.95 | | 5.95 | 1687.25 | *** |  | 312656.68 | | | 312656.68 | 123.38 | *** |  |
| Accession x Treatment | 15 | 8799.84 | 586.66 | 3.94 | *** |  | 0.73 | | 0.05 | 13.74 | *** |  | 134472.49 | | | 8964.83 | 3.54 | *** |  |
| Residuals | 121 | 18024.14 | 148.96 |  |  |  | 0.43 | | 0.00 |  |  |  | 306621.20 | | | 2534.06 |  |  | |

Two-way ANOVA testing effects of accession, treatment, and their interaction on germination timing indices (MGT, IVG, DDS₅₀). FGP analyzed separately (Fig. 2B). MGT, IVG and DDS₅₀ describe related but non-identical components of germination timing and speed and were therefore analyzed separately; IVG and DDS₅₀ are jointly visualized in Fig. 2D, whereas MGT is shown independently.

Df = degrees of freedom; SS = sum of squares; MS = mean square.

Significance codes: *p* < 0.05 (*), *p* < 0.01 (**), *p* < 0.001 (***), ns = not significant.

**Table S5.** Mean germination parameters (± SD) for 16 *L. angustifolius* accessions under non-scarified (NS) and scarified (S) treatments, and principal component scores (PC1–PC2) under non-scarified (NS) conditions.

| **Accession** | **Dormancy Group** | **Treatment NS** | | | | | | | | **Treatment S** | | | | | |
| --- | --- | --- | --- | --- | --- | --- | --- | --- | --- | --- | --- | --- | --- | --- | --- |
|  |  | **MGT** | **DDS_50_** | **IVG** | **FGP %** | **PD %** | **PC1** | **PC2** | **MGT** | | **DDS_50_** | **IVG** | **FGP %** | **PD %** |  |
| LUP-0020 | Low | 11.96 ± 11.07 | 59.80 ± 55.35 | 0.05 ± 0.05 | 100.0 ± 0.0 | 0.0 ± 0.0 | 0.858 | 0.379 | 0.40 ± 0.00 | | 2.00 ± 0.00 | 0.50 ± 0.00 | 100.0 ± 0.0 | 0.0 ± 0.0 |  |
| LUP-0024 | Low | 10.88 ± 6.06 | 43.50 ± 24.26 | 0.04 ± 0.05 | 80.0 ± 44.7 | 20.0 ± 44.7 | 0.570 | -0.422 | 0.40 ± 0.00 | | 2.00 ± 0.00 | 0.50 ± 0.00 | 100.0 ± 0.0 | 0.0 ± 0.0 |  |
| LUP-0249 | Low | 5.68 ± 3.78 | 28.40 ± 18.92 | 0.05 ± 0.03 | 100.0 ± 0.0 | 0.0 ± 0.0 | 1.367 | 0.323 | 1.16 ± 0.17 | | 5.80 ± 0.84 | 0.18 ± 0.02 | 100.0 ± 0.0 | 0.0 ± 0.0 |  |
| LUP-0251 | Low | 12.62 ± 9.51 | 50.50 ± 38.04 | 0.05 ± 0.05 | 80.0 ± 44.7 | 20.0 ± 44.7 | 0.445 | -0.415 | 0.40 ± 0.00 | | 2.00 ± 0.00 | 0.50 ± 0.00 | 100.0 ± 0.0 | 0.0 ± 0.0 |  |
| LUP-0254 | Low | 4.72 ± 4.47 | 23.60 ± 22.33 | 0.07 ± 0.05 | 100.0 ± 0.0 | 0.0 ± 0.0 | 1.521 | 0.183 | 0.40 ± 0.00 | | 2.00 ± 0.00 | 0.50 ± 0.00 | 100.0 ± 0.0 | 0.0 ± 0.0 |  |
| LUP-0293 | Low | 0.40 ± 0.00 | 2.00 ± 0.00 | 0.50 ± 0.00 | 100.0 ± 0.0 | 0.0 ± 0.0 | 3.266 | -2.280 | 0.40 ± 0.00 | | 2.00 ± 0.00 | 0.50 ± 0.00 | 100.0 ± 0.0 | 0.0 ± 0.0 |  |
| LUP-0008 | Intermediate | 23.88 ± 16.79 | 119.40 ± 83.95 | 0.02 ± 0.02 | 100.0 ± 0.0 | 0.0 ± 0.0 | -0.201 | 0.641 | 0.40 ± 0.00 | | 2.00 ± 0.00 | 0.50 ± 0.00 | 100.0 ± 0.0 | 0.0 ± 0.0 |  |
| LUP-0023 | Intermediate | 19.96 ± 14.85 | 99.80 ± 74.26 | 0.02 ± 0.03 | 100.0 ± 0.0 | 0.0 ± 0.0 | 0.136 | 0.575 | 0.40 ± 0.00 | | 2.00 ± 0.00 | 0.50 ± 0.00 | 100.0 ± 0.0 | 0.0 ± 0.0 |  |
| LUP-0255 | Intermediate | 12.00 ± 11.54 | 60.00 ± 57.72 | 0.04 ± 0.03 | 100.0 ± 0.0 | 0.0 ± 0.0 | 0.815 | 0.447 | 0.60 ± 0.45 | | 3.00 ± 2.24 | 0.43 ± 0.16 | 100.0 ± 0.0 | 0.0 ± 0.0 |  |
| LUP-0257 | Intermediate | 13.88 ± 8.67 | 69.40 ± 43.33 | 0.02 ± 0.01 | 100.0 ± 0.0 | 0.0 ± 0.0 | 0.611 | 0.554 | 0.80 ± 0.55 | | 4.00 ± 2.74 | 0.36 ± 0.20 | 100.0 ± 0.0 | 0.0 ± 0.0 |  |
| LUP-0028 | High | 38.84 ± 22.53 | 194.20 ± 112.65 | 0.03 ± 0.05 | 100.0 ± 0.0 | 0.0 ± 0.0 | -1.368 | 0.694 | 0.40 ± 0.00 | | 2.00 ± 0.00 | 0.50 ± 0.00 | 100.0 ± 0.0 | 0.0 ± 0.0 |  |
| LUP-0032 | High | 50.00 ± 43.50 | 150.00 ± 130.50 | 0.01 ± 0.01 | 60.0 ± 54.8 | 40.0 ± 54.8 | -2.339 | -1.006 | 0.40 ± 0.00 | | 2.00 ± 0.00 | 0.50 ± 0.00 | 100.0 ± 0.0 | 0.0 ± 0.0 |  |
| LUP-0042 | High | 35.52 ± 20.61 | 177.60 ± 103.07 | 0.02 ± 0.03 | 100.0 ± 0.0 | 0.0 ± 0.0 | -1.131 | 0.721 | 0.60 ± 0.45 | | 3.00 ± 2.24 | 0.43 ± 0.16 | 100.0 ± 0.0 | 0.0 ± 0.0 |  |
| LUP-0248 | High | 27.48 ± 12.73 | 137.40 ± 63.65 | 0.01 ± 0.01 | 100.0 ± 0.0 | 0.0 ± 0.0 | -0.518 | 0.719 | 0.40 ± 0.00 | | 2.00 ± 0.00 | 0.50 ± 0.00 | 100.0 ± 0.0 | 0.0 ± 0.0 |  |
| LUP-0253 | High | 27.76 ± 15.86 | 138.80 ± 79.30 | 0.02 ± 0.02 | 100.0 ± 0.0 | 0.0 ± 0.0 | -0.517 | 0.6800 | 0.40 ± 0.00 | | 2.00 ± 0.00 | 0.50 ± 0.00 | 100.0 ± 0.0 | 0.0 ± 0.0 |  |
| LUP-0256 | High | 61.67 ± 70.71 | 185.00 ± 212.13 | 0.02 ± 0.02 | 40.0 ± 54.8 | 60.0 ± 54.8 | -3.515 | -1.794 | 0.40 ± 0.00 | | 2.00 ± 0.00 | 0.50 ± 0.00 | 100.0 ± 0.0 | 0.0 ± 0.0 |  |

Traits include: MGT (mean germination time, days), DDS₅₀ (days to 50 % germination), IVG (germination velocity index), FGP (final germination percentage, %), and PD (physical dormancy, % = 100 − FGP_NS_). Values correspond to accession-level means ± SD computed from seed-level data. Dormancy groups (Low, Intermediate, High) were assigned on multivariate analyses of germination parameters (PCA and hierarchical clustering) under NS conditions (see Fig. S2). Color coding applies to NS treatment values and reflects dormancy groups assigned under NS conditions (Low = green, Intermediate = orange, High = red). PCA scores (PC1, PC2) represent accession coordinates in the principal-component space under NS conditions.

**Table S6**. Global and treatment-specific Pearson correlations among germination parameters (FGP, MGT, IVG, DDS₅₀) calculated across *L. angustifolius* accessions.

| **Trait** | **Treatment** | **MGT** | | | **IVG** | | | **DDS_50_** | | |
| --- | --- | --- | --- | --- | --- | --- | --- | --- | --- | --- |
|  |  | **r** | ***p*-value** | **Significance** | **r** | ***p*-value** | **Significance** | **r** | ***p*-value** | **Significance** |
| **FGP** | **Global** | -0.687 | 0.0000 | *** | 0.357 | 0.0448 | * | -0.459 | 0.0082 | ** |
|  | **NS** | -0.664 | 0.0050 | ** | 0.164 | 0.5448 | ns | -0.331 | 0.2105 | ns |
|  | **S** | NA | NA | NA | NA | NA | NA | NA | NA | NA |
| **MGT** | **Global** |  |  |  | -0.727 | <0.0001 | *** | 0.955 | <0.0001 | *** |
|  | **NS** |  |  |  | -0.437 | 0.0908 | ns | 0.916 | <0.0001 | *** |
|  | **S** |  |  |  | -0.996 | <0.0001 | *** | 1.000 | <0.0001 | *** |
| **IVG** | **Global** |  |  |  |  |  |  | -0.788 | <0.0001 | *** |
|  | **NS** |  |  |  |  |  |  | -0.505 | 0.0459 | * |
|  | **S** |  |  |  |  |  |  | -0.996 | <0.0001 | *** |

Trait-specific correlations are reported for accession-level mean values under non-scarified (NS) and scarified (S) treatments. Traits include: FGP (final germination percentage, %), MGT (mean germination time, days), IVG (germination velocity index), DDS₅₀ (days to 50 % germination). Global correlations were computed pooling accession means across treatments, whereas treatment-specific correlations were computed separately for NS and S conditions. NA indicates correlations not computed due to lack of variability within a treatment. Significance codes: *** *p* < 0.001, ** *p* < 0.01, * *p* < 0.05, ns = not significant.

**Table S7.** Variance explained by principal components (PCA) and loadings of germination variables under non-scarified (NS) conditions.

| **Component** | **% Variance** | **% Cumulative** | **IVG** | **MGT** | **DDS_50_** | **FGP** |
| --- | --- | --- | --- | --- | --- | --- |
| PC1 | 64.5 | 64.5 | 0.389 | −0.605 | −0.560 | 0.411 |
| PC2 | 22.2 | 86.7 | −0.676 | −0.147 | 0.199 | 0.694 |
| PC3 | 13.1 | 99.8 |  |  |  |  |
| PC4 | 0.2 | 100.0 |  |  |  |  |

PCA was conducted on standardized accession-level means for IVG (germination velocity index), MGT (mean germination time), DDS₅₀ (days to 50 % germination), and FGP (final germination percentage). PC1 and PC2 together accounted for 86.7 % of total variance.

Loadings represent variable contributions; higher absolute values indicate stronger influence. The sign of loadings is arbitrary.

**Table S8.** Predicted means and confidence intervals from GLMM models for germination, cotyledon emergence, and early establishment performance.

| **Model term** | **Germination** | | |  | **Cotyledon** | | |  | **Early establishment** | | |
| --- | --- | --- | --- | --- | --- | --- | --- | --- | --- | --- | --- |
|  | **Estimate** | **CI Lower** | **CI Upper** |  | **Estimate** | **CI Lower** | **CI Upper** |  | **Estimate** | **CI Lower** | **CI Upper** |
| (Intercept) | 0.75 | 0.32 | 1.18 |  | 0.18 | -0.28 | 0.63 |  | -0.20 | -0.66 | 0.27 |
| *L. angustifolius* | -2.43 | -3.04 | -1.82 |  | -2.19 | -2.84 | -1.53 |  | -1.87 | -2.54 | -1.19 |
| *L. luteus* | -0.52 | -1.12 | 0.09 |  | -2.42 | -3.07 | -1.77 |  | -2.15 | -2.82 | -1.48 |
| COPMO | 1.12 | 0.81 | 1.43 |  | 0.63 | 0.42 | 0.84 |  | 0.59 | 0.39 | 0.78 |
| COPSA | 0.72 | 0.43 | 1.00 |  | 0.96 | 0.73 | 1.18 |  | 0.88 | 0.68 | 1.08 |
| MBG3 | 1.21 | 0.89 | 1.53 |  | 1.42 | 1.16 | 1.67 |  | 1.38 | 1.16 | 1.60 |
| MEC | 0.83 | 0.50 | 1.17 |  | 0.87 | 0.61 | 1.14 |  | -0.03 | -0.24 | 0.18 |
| Treatments | 2.19 | 2.08 | 2.29 |  | 1.49 | 1.40 | 1.57 |  | 1.33 | 1.24 | 1.41 |
| *L. angustifolius*: COPMO | -0.22 | -0.59 | 0.15 |  | 0.65 | 0.36 | 0.94 |  | 0.63 | 0.35 | 0.91 |
| *L. luteus*: COPMO | -0.07 | -0.49 | 0.35 |  | 0.95 | 0.64 | 1.25 |  | 0.92 | 0.63 | 1.21 |
| *L. angustifolius:* COPSA | -0.03 | -0.38 | 0.32 |  | -0.02 | -0.32 | 0.28 |  | 0.09 | -0.19 | 0.37 |
| *L. luteus*: COPSA | 0.05 | -0.34 | 0.44 |  | 0.76 | 0.45 | 1.07 |  | 0.82 | 0.53 | 1.12 |
| *L.angustifolius:* MBG3 | -0.14 | -0.52 | 0.24 |  | 0.29 | -0.03 | 0.61 |  | 0.38 | 0.08 | 0.68 |
| *L. luteus:* MBG3 | -0.14 | -0.57 | 0.28 |  | 1.53 | 1.18 | 1.88 |  | 1.53 | 1.21 | 1.86 |
| *L. angustifolius:* MEC | -0.07 | -0.48 | 0.34 |  | 0.61 | 0.27 | 0.96 |  | 0.80 | 0.48 | 1.12 |
| *L. luteus:* MEC | -0.43 | -0.88 | 0.01 |  | 1.29 | 0.93 | 1.65 |  | 2.27 | 1.94 | 2.60 |

Estimates are presented by *Lupinus* species and soil type, calculated across accessions and pooled across scarification treatments (non-scarified and scarified). The (Intercept) corresponds to the reference levels in the model (species = *L. albus*; soil = BV; and the reference level of the scarification factor). Values correspond to GLMM-derived predicted means (logit scale) for each trait, with 95 % confidence intervals (CIs).

**Table S9**. Observed germination, cotyledon emergence, and early establishment rates (%) by species, soil type, and scarification treatment.

| **Species** | **SOIL** | **Treatment NS** | | | |  | **Treatment S** | | | |
| --- | --- | --- | --- | --- | --- | --- | --- | --- | --- | --- |
|  |  | **N** | **Germination** | **Cotyledon** | **Early establishment** |  | **N** | **Germination** | **Cotyledon** | **Early establishment** |
| *L. albus* | BV | 340 | 82.94 | 70.88 | 57.35 |  | 855 | 87.60 | 76.61 | 70.29 |
| *L. albus* | COPMO | 340 | 85.00 | 81.47 | 72.65 |  | 855 | 98.01 | 84.91 | 78.71 |
| *L. albus* | COPSA | 340 | 87.06 | 75.59 | 67.94 |  | 855 | 94.04 | 92.28 | 86.67 |
| *L. albus* | MBG3 | 340 | 97.06 | 94.12 | 90.59 |  | 855 | 93.80 | 90.41 | 85.96 |
| *L. albus* | MEC | 220 | 86.82 | 73.64 | 39.09 |  | 495 | 93.33 | 89.49 | 70.51 |
| *L. angustifolius* | BV | 300 | 8.67 | 2.00 | 1.67 |  | 750 | 65.60 | 44.80 | 40.80 |
| *L. angustifolius* | COPMO | 300 | 7.33 | 6.67 | 5.67 |  | 750 | 88.53 | 78.80 | 72.53 |
| *L. angustifolius* | COPSA | 300 | 11.00 | 4.33 | 4.33 |  | 750 | 82.13 | 70.00 | 66.00 |
| *L. angustifolius* | MBG3 | 300 | 7.67 | 7.67 | 7.67 |  | 750 | 92.27 | 89.87 | 86.80 |
| *L. angustifolius* | MEC | 200 | 9.00 | 8.00 | 7.50 |  | 450 | 76.44 | 72.67 | 44.00 |
| *L. luteus* | BV | 320 | 74.38 | 36.25 | 33.75 |  | 810 | 82.35 | 31.73 | 28.64 |
| *L. luteus* | COPMO | 320 | 80.94 | 45.94 | 43.12 |  | 810 | 93.58 | 63.58 | 58.15 |
| *L. luteus* | COPSA | 320 | 73.44 | 47.19 | 43.75 |  | 810 | 93.46 | 66.30 | 62.35 |
| *L. luteus* | MBG3 | 320 | 77.50 | 67.81 | 63.44 |  | 810 | 95.06 | 83.33 | 80.37 |
| *L. luteus* | MEC | 200 | 85.50 | 51.00 | 49.00 |  | 450 | 77.56 | 50.44 | 45.78 |

Values represent the percentages of individuals showing each trait for three *Lupinus* species across five soils and two treatments (non-scarified = NS; scarified = S). N represents the number of seeds sown per species × soil × treatment combination.

**Table S10.** Germination, cotyledon emergence, and early establishment rates (%) in *Lupinus* species across biological types and scarification treatments, averaged across soils.

| **Specie** | **Type** | **Treatment NS** | | | |  | **Treatment S** | | | |
| --- | --- | --- | --- | --- | --- | --- | --- | --- | --- | --- |
|  |  | **N** | **Germination** | **Cotyledon** | **Early establishment** |  | **N** | **Germination** | **Cotyledon** | **Early establishment** |
| ***L. albus*** | Cultivar | 100 | 82.00 | 64.00 | 58.00 |  | 225 | 96.44 | 89.78 | 88.89 |
| ***L. angustifolius*** | Cultivar | 100 | 93.00 | 49.00 | 45.00 |  | 225 | 90.67 | 72.00 | 68.00 |
| ***L. luteus*** | Cultivar | 100 | 98.00 | 64.00 | 59.00 |  | 225 | 84.89 | 59.56 | 52.00 |
| **Total** | | **300** | **91.00** | **59.00** | **54.00** |  | **675** | **90.67** | **73.78** | **69.63** |
| ***L. albus*** | Landrace | 1300 | 88.69 | 81.08 | 68.31 |  | 3225 | 93.09 | 85.92 | 78.23 |
| ***L. angustifolius*** | Landrace | 100 | 0.00 | 0.00 | 0.00 |  | 225 | 64.89 | 53.33 | 49.33 |
| ***L. luteus*** | Landrace | 920 | 77.72 | 43.80 | 41.09 |  | 2310 | 90.17 | 61.17 | 57.01 |
| **Total** | | **2320** | **55.47** | **41.63** | **36.47** |  | **5760** | **82.72** | **66.81** | **61.52** |
| ***L. albus*** | Wild | 180 | 85.00 | 77.22 | 67.22 |  | 465 | 93.76 | 88.82 | 80.86 |
| ***L. angustifolius*** | Wild | 1200 | 2.42 | 2.42 | 2.33 |  | 3000 | 81.93 | 72.37 | 64.33 |
| ***L. luteus*** | Wild | 460 | 73.48 | 57.83 | 54.35 |  | 1155 | 88.92 | 57.49 | 54.63 |
| **Total** | | **1840** | **53.63** | **45.82** | **41.30** |  | **4620** | **88.20** | **72.89** | **66.61** |

Data are disaggregated by species and biological type (cultivar, landrace, wild) under non-scarified (NS) and scarified (S) treatments, pooled across soils. Values are percentages of individuals showing each trait. N represents the number of seeds sown per species × biological type × treatment combination.

**Table S11.** PCA loadings for PC1 and PC2 based on seedling traits. Eigenvalues, variance explained, and cumulative variance of the principal components of seedling performance.

| **Variable** | **PC1** | **PC2** |
| --- | --- | --- |
| **Germination** | 0.5285 | −0.8488 |
| **Cotyledon** | 0.6015 | 0.3594 |
| **Early establishment** | 0.5991 | 0.3879 |
| **SD (√ eigenvalue)** | 1.5993 | 0.6297 |
| **% Variance** | 85.26 | 13.22 |
| **% Cumulative** | 85.26 | 98.48 |

Loadings indicate the contribution of germination, cotyledon emergence, and early establishment to each principal component. PC1 captures overall seedling establishment performance, while PC2 reflects partial decoupling among early seedling traits across soils.

**Table S12.** Top five *Lupinus* accessions by early seedling establishment (%) in each soil.

| **Genotype** | **Species** | **Soil** | **Early seedling establishment (%)** |
| --- | --- | --- | --- |
| LUP-0219 | *L. albus* | BV | 90.77 |
| LUP-0222 | *L. albus* | BV | 90.00 |
| LUP-0236 | *L. albus* | BV | 76.25 |
| LUP-0242 | *L. albus* | BV | 76.25 |
| LUP-0237 | *L. albus* | BV | 75.38 |
| LUP-0233 | *L. albus* | COPMO | 98.75 |
| LUP-0228 | *L. albus* | COPMO | 97.50 |
| LUP-0280 | *L. luteus* | COPMO | 97.50 |
| LUP-0074 | *L. luteus* | COPMO | 91.25 |
| LUP-0236 | *L. albus* | COPMO | 90.00 |
| LUP-0236 | *L. albus* | COPSA | 98.75 |
| LUP-0233 | *L. albus* | COPSA | 97.50 |
| LUP-0228 | *L. albus* | COPSA | 96.25 |
| LUP-0226 | *L. albus* | COPSA | 95.00 |
| LUP-0074 | *L. luteus* | COPSA | 93.75 |
| LUP-0074 | *L. luteus* | MBG3 | 100.00 |
| LUP-0280 | *L. luteus* | MBG3 | 100.00 |
| LUP-0232 | *L. albus* | MBG3 | 98.46 |
| LUP-0279 | *L. luteus* | MBG3 | 97.50 |
| LUP-0235 | *L. albus* | MBG3 | 96.92 |
| LUP-0237 | *L. albus* | MEC | 84.62 |
| LUP-0293 | *L. angustifolius* | MEC | 83.08 |
| LUP-0294 | *L. albus* | MEC | 83.08 |
| LUP-0290 | *L. albus* | MEC | 76.92 |
| LUP-0241 | *L. albus* | MEC | 70.77 |

Accessions are ranked by early seedling establishment within each soil. High-ranking accessions were predominantly *L. albus* and *L. luteus* in MBG3, COPMO and COPSA, whereas only a limited subset of *L. angustifolius* accessions achieved comparably high establishment in MEC.

**Table S13.** Pearson’s correlation coefficients between dormancy (DDS₅₀) and early seedling establishment in *L. angustifolius* across soils.

| **Soil** | **n** | **Pearson’s r** | ***p*-value** |
| --- | --- | --- | --- |
| BV | 14 | 0.16 | 0.57 |
| COPMO | 14 | 0.02 | 0.94 |
| COPSA | 14 | 0.10 | 0.72 |
| MBG3 | 14 | −0.24 | 0.41 |
| MEC | 10 | −0.33 | 0.35 |

Sample size (n), Pearson’s correlation coefficients (r), and corresponding p-values are shown for each soil. No significant correlations were detected between dormancy (DDS₅₀) and early seedling establishment in any soil (all *p* > 0.3).

**Table S14**. Post hoc contrasts for the effects of substrate pH and days after sowing (DAS) on germination, early establishment, and seedling growth traits in *Lupinus* species.

1. Germination and early establishment — Tukey-adjusted pairwise comparisons of pH effects within each DAS level.

| **DAS** | **Contrast** | **Germination OR (SE), z, p** | **Establishment OR (SE), z, p** |
| --- | --- | --- | --- |
| **7** | 5.5 vs 7.0 | 0.79 (0.12), –1.62, 0.105 | 0.55 (0.06), –5.38, < 0.0001 |
| **7** | 5.5 vs 8.5 | — | — |
| **7** | 7.0 vs 8.5 | — | — |
| **14** | 5.5 vs 7.0 | 0.78 (0.12), –1.64, 0.229 | 0.87 (0.11), –1.14, 0.488 |
| **14** | 5.5 vs 8.5 | 0.92 (0.13), –0.58, 0.830 | 1.16 (0.14), 1.22, 0.441 |
| **14** | 7.0 vs 8.5 | 1.18 (0.18), 1.06, 0.538 | 1.34 (0.17), 2.36, 0.048 |

1. Seedling growth traits — Tukey’s HSD tests for main effects of pH and DAS (two-way ANOVA).

| **Trait** | **Source** | **Contrast** | **Mean diff. (mm)** | **95 % CI** | **p-adj** |
| --- | --- | --- | --- | --- | --- |
| **Hypocotyl length** | pH | 5.5–7.0 | −2.27 | −4.20 – −0.35 | 0.0158 |
|  | pH | 5.5–8.5 | 7.52 | 5.50 – 9.55 | < 0.0001 |
|  | pH | 7.0–8.5 | 9.8 | 7.81 – 11.78 | < 0.0001 |
|  | DAS | 7–14 | −13.98 | −15.33 – −12.63 | < 0.0001 |
| **Epicotyl length** | pH | 5.5–7.0 | −0.79 | −1.55 – −0.03 | 0.0379 |
|  | pH | 5.5–8.5 | 1.73 | 0.93 – 2.52 | < 0.0001 |
|  | pH | 7.0–8.5 | 2.52 | 1.74 – 3.30 | < 0.0001 |
|  | DAS | 7–14 | −12.19 | −12.72 – −11.66 | < 0.0001 |
| **Total seedling length** | pH | 5.5–7.0 | −3.06 | −5.30 – −0.82 | 0.0039 |
|  | pH | 5.5–8.5 | 9.25 | 6.89 – 11.61 | < 0.0001 |
|  | pH | 7.0–8.5 | 12.31 | 10.00 – 14.62 | < 0.0001 |
|  | DAS | 7–14 | −26.17 | −27.74 – −24.60 | < 0.0001 |
| **Hypocotyl-to-epicotyl ratio** | pH | 5.5–7.0 | 2024.42 | 205.45 – 3843.39 | 0.0247 |
|  | pH | 7.0–8.5 | −2281.99 | −4158.65 – −405.34 | 0.0122 |
|  | DAS | 7–14 | 16368.47 | 15090.89 – 17646.05 | < 0.0001 |

Odds ratios (OR) (standard errors, SE), z-values, and p-values are shown for germination and early establishment (binomial GLMMs). Mean differences ± 95 % CIs and Tukey-adjusted p-values are reported for continuous growth traits. Pairwise pH contrasts (5.5–7.0, 5.5–8.5, 7.0–8.5) are reported within each DAS level in panel (a), and main-effect contrasts for pH and DAS (7–14 days) are reported for growth traits in panel (b). Non-estimable contrasts (“—”) result from missing observations at pH 8.5 × DAS 7. All models met homoscedasticity (Levene’s p > 0.05) and normality (Shapiro–Wilk p > 0.05).

**Table S15.** Cluster membership of 48 *Lupinus* accessions based on hierarchical clustering of standardized phenotypic traits.

| **Accession** | **Cluster** | **Species** | **Status** |
| --- | --- | --- | --- |
| **LUP-0008** | 1 | *L. angustifolius* | Wild |
| **LUP-0024** | 1 | *L. angustifolius* | Wild |
| **LUP-0032** | 1 | *L. angustifolius* | Wild |
| **LUP-0257** | 1 | *L. angustifolius* | Wild |
| **LUP-0291** | 1 | *L. angustifolius* | Wild |
| **LUP-0295** | 1 | *L. luteus* | Cultivar |
| **LUP-0074** | 2 | *L. luteus* | Wild |
| **LUP-0219** | 2 | *L. albus* | Landrace |
| **LUP-0222** | 2 | *L. albus* | Landrace |
| **LUP-0226** | 2 | *L. albus* | Landrace |
| **LUP-0227** | 2 | *L. albus* | Landrace |
| **LUP-0228** | 2 | *L. albus* | Wild |
| **LUP-0231** | 2 | *L. albus* | Landrace |
| **LUP-0232** | 2 | *L. albus* | Landrace |
| **LUP-0233** | 2 | *L. albus* | Landrace |
| **LUP-0235** | 2 | *L. albus* | Wild |
| **LUP-0236** | 2 | *L. albus* | Landrace |
| **LUP-0237** | 2 | *L. albus* | Landrace |
| **LUP-0238** | 2 | *L. albus* | Landrace |
| **LUP-0241** | 2 | *L. albus* | Landrace |
| **LUP-0242** | 2 | *L. albus* | Landrace |
| **LUP-0245** | 2 | *L. albus* | Landrace |
| **LUP-0261** | 2 | *L. luteus* | Landrace |
| **LUP-0266** | 2 | *L. luteus* | Landrace |
| **LUP-0269** | 2 | *L. luteus* | Landrace |
| **LUP-0279** | 2 | *L. luteus* | Wild |
| **LUP-0280** | 2 | *L. luteus* | Landrace |
| **LUP-0290** | 2 | *L. albus* | Landrace |
| **LUP-0294** | 2 | *L. albus* | Cultivar |
| **LUP-0264** | 3 | *L. luteus* | Landrace |
| **LUP-0265** | 3 | *L. luteus* | Landrace |
| **LUP-0271** | 3 | *L. luteus* | Landrace |
| **LUP-0273** | 3 | *L. luteus* | Landrace |
| **LUP-0274** | 3 | *L. luteus* | Wild |
| **LUP-0278** | 3 | *L. luteus* | Wild |
| **LUP-0281** | 3 | *L. luteus* | Landrace |
| **LUP-0293** | 3 | *L. angustifolius* | Cultivar |
| **LUP-0028** | 4 | *L. angustifolius* | Wild |
| **LUP-0248** | 4 | *L. angustifolius* | Wild |
| **LUP-0253** | 4 | *L. angustifolius* | Landrace |
| **LUP-0255** | 4 | *L. angustifolius* | Wild |
| **LUP-0256** | 4 | *L. angustifolius* | Wild |
| **LUP-0020** | 5 | *L. angustifolius* | Wild |
| **LUP-0249** | 5 | *L. angustifolius* | Wild |
| **LUP-0251** | 5 | *L. angustifolius* | Wild |
| **LUP-0254** | 5 | *L. angustifolius* | Wild |
| **LUP-0267** | 5 | *L. luteus* | Wild |
| **LUP-0268** | 5 | *L. luteus* | Landrace |

Accessions were grouped into five clusters (Fig. 10A) based on standardized phenotypic traits related to germination, early seedling establishment, and seedling growth measured across experiments. Cluster composition reflects both species-specific and mixed-species phenotypic response patterns. Species identity and domestication status (Wild, Landrace, Cultivar) are provided for descriptive purposes only and are not used as clustering variables. Cluster membership summarizes phenotypic similarity under the tested conditions and does not imply genetic uniformity within accessions or a causal role of domestication status.

**Table S16**. Principal component loadings for 48 *Lupinus* accessions based on scaled phenotypic traits (first five PCs).

| **Variable** | **PC1** | **PC2** | **PC3** | **PC4** | **PC5** | **PC1_contribution** | **PC2_contribution** | **PC3_contribution** | **Communality** |
| --- | --- | --- | --- | --- | --- | --- | --- | --- | --- |
| **Germination (BV)** | 0.239 | 0.145 | -0.067 | -0.047 | 0.243 | 5.73 | 2.10 | 0.45 | 8.28 |
| **Germination (COPMO)** | 0.260 | 0.099 | -0.104 | -0.137 | 0.117 | 6.73 | 0.99 | 1.08 | 8.80 |
| **Germination (COPSA)** | 0.261 | 0.100 | -0.061 | -0.050 | 0.071 | 6.83 | 0.99 | 0.37 | 8.19 |
| **Germination (MBG3)** | 0.270 | 0.129 | -0.113 | -0.087 | 0.075 | 7.30 | 1.66 | 1.28 | 10.23 |
| **Germination (MEC)** | 0.225 | 0.091 | -0.215 | -0.164 | -0.327 | 5.06 | 0.83 | 4.63 | 10.52 |
| **Cotyledon emergence (BV)** | 0.237 | -0.173 | 0.228 | 0.078 | 0.128 | 5.60 | 2.99 | 5.20 | 13.78 |
| **Cotyledon emergence (COPMO)** | 0.246 | -0.189 | 0.109 | 0.082 | 0.007 | 6.04 | 3.58 | 1.18 | 10.80 |
| **Cotyledon emergence (COPSA)** | 0.272 | -0.136 | 0.143 | 0.024 | 0.109 | 7.40 | 1.85 | 2.04 | 11.29 |
| **Cotyledon emergence (MBG3)** | 0.289 | -0.017 | -0.049 | 0.000 | 0.028 | 8.36 | 0.03 | 0.24 | 8.62 |
| **Cotyledon emergence (MEC)** | 0.234 | -0.069 | -0.146 | -0.018 | -0.470 | 5.49 | 0.47 | 2.12 | 8.09 |
| **Establishment (BV)** | 0.237 | -0.168 | 0.250 | 0.105 | 0.142 | 5.62 | 2.81 | 6.27 | 14.70 |
| **Establishment (COPMO)** | 0.245 | -0.175 | 0.126 | 0.106 | -0.005 | 5.98 | 3.06 | 1.59 | 10.63 |
| **Establishment (COPSA)** | 0.265 | -0.122 | 0.163 | 0.048 | 0.117 | 7.00 | 1.49 | 2.66 | 11.15 |
| **Establishment (MBG3)** | 0.284 | -0.025 | -0.031 | 0.030 | 0.027 | 8.03 | 0.06 | 0.10 | 8.19 |
| **Establishment (MEC)** | 0.209 | 0.083 | -0.246 | -0.009 | -0.422 | 4.38 | 0.69 | 6.03 | 11.10 |
| **IVG (Exp. 1)** | 0.078 | 0.108 | -0.265 | -0.063 | -0.091 | 0.61 | 1.17 | 7.02 | 8.80 |
| **MGT (Exp. 1)** | -0.009 | -0.035 | 0.378 | -0.540 | -0.196 | 0.01 | 0.12 | 14.29 | 14.43 |
| **DDS_50_ (Exp. 1)** | -0.017 | -0.041 | 0.415 | -0.519 | -0.195 | 0.03 | 0.17 | 17.18 | 17.38 |
| **Establishment (pH 5.5)** | -0.023 | -0.417 | -0.018 | 0.156 | -0.172 | 0.05 | 17.37 | 0.03 | 17.46 |
| **Establishment (pH 7.0)** | -0.100 | -0.215 | -0.234 | -0.288 | 0.316 | 1.00 | 4.60 | 5.48 | 11.08 |
| **Total seedling length (pH 5.5)** | -0.046 | -0.428 | -0.083 | 0.095 | -0.190 | 0.21 | 18.31 | 0.69 | 19.21 |
| **Total seedling length (pH 7.0)** | -0.108 | -0.323 | -0.195 | -0.176 | 0.075 | 1.17 | 10.44 | 3.79 | 15.40 |
| **Germination (pH 5.5)** | 0.071 | -0.196 | -0.237 | -0.256 | 0.196 | 0.50 | 3.84 | 5.63 | 9.97 |
| **Germination (pH 7.0)** | 0.042 | -0.185 | -0.319 | -0.344 | 0.191 | 0.18 | 3.42 | 10.15 | 13.75 |
| **Integrated selection index (ISI)** | -0.084 | -0.412 | -0.072 | 0.083 | -0.129 | 0.71 | 16.94 | 0.52 | 18.18 |

Loadings indicate the contribution of each trait to the first five principal components obtained from the principal component analysis (PCA shown in Fig. 10B) performed on standardized data. Trait contributions (%) were calculated as squared loadings × 100 for each PC. Communality is reported for PC1–PC3 and represents the proportion of variance in each trait explained by these components. Traits include germination, cotyledon emergence, and establishment measured across soil environments (Experiment 2); germination dynamics from the physical dormancy assay (Experiment 1); and germination, establishment, and seedling growth traits measured under substrate pH gradients (Experiment 3). Germination = proportion of seeds reaching score ≥ 1; Cotyledon emergence = score ≥ 3; Establishment = score = 5 in soil assays, or proportion of seedlings reaching total length ≥ 30 mm in the pH experiment. The Integrated Selection Index (ISI), derived from Experiment 3 variables, is included here as an auxiliary trait for descriptive multivariate comparison. Variance explained by the first five PCs: PC1 = 42.0%, PC2 = 17.5%, PC3 = 8.8%, PC4 = 7.9%, and PC5 = 4.6%.

**Table S17**. Principal component scores (PC1–PC3) and cluster membership of 48 *Lupinus* accessions.

| **Accession** | **Cluster** | **PC1** | **PC2** | **PC3** | **Species** | **Status** |
| --- | --- | --- | --- | --- | --- | --- |
| **LUP-0008** | 1 | 0.205 | -0.770 | 1.565 | *L. angustifolius* | Wild |
| **LUP-0020** | 5 | -5.373 | -0.145 | 0.523 | *L. angustifolius* | Wild |
| **LUP-0024** | 1 | -0.026 | -0.230 | -0.887 | *L. angustifolius* | Wild |
| **LUP-0028** | 4 | -3.982 | -0.438 | 3.120 | *L. angustifolius* | Wild |
| **LUP-0032** | 1 | -1.182 | -0.252 | 1.413 | *L. angustifolius* | Wild |
| **LUP-0074** | 2 | 2.720 | -0.829 | 0.413 | *L. luteus* | Wild |
| **LUP-0219** | 2 | 2.678 | -1.546 | 0.410 | *L. albus* | Landrace |
| **LUP-0222** | 2 | 2.215 | -1.025 | 0.653 | *L. albus* | Landrace |
| **LUP-0226** | 2 | 2.236 | -1.315 | 0.120 | *L. albus* | Landrace |
| **LUP-0227** | 2 | 1.265 | 0.394 | 1.227 | *L. albus* | Landrace |
| **LUP-0228** | 2 | 1.909 | -3.749 | -0.411 | *L. albus* | Wild |
| **LUP-0231** | 2 | 2.053 | 2.106 | 1.004 | *L. albus* | Landrace |
| **LUP-0232** | 2 | 3.150 | -1.814 | -0.608 | *L. albus* | Landrace |
| **LUP-0233** | 2 | 3.745 | 0.465 | 1.124 | *L. albus* | Landrace |
| **LUP-0235** | 2 | 2.929 | -2.321 | -1.285 | *L. albus* | Wild |
| **LUP-0236** | 2 | 3.502 | -2.972 | -0.055 | *L. albus* | Landrace |
| **LUP-0237** | 2 | 4.293 | -0.981 | 0.106 | *L. albus* | Landrace |
| **LUP-0238** | 2 | 1.405 | -1.926 | -0.711 | *L. albus* | Landrace |
| **LUP-0241** | 2 | 3.734 | -0.156 | -0.223 | *L. albus* | Landrace |
| **LUP-0242** | 2 | 2.923 | -0.283 | 0.181 | *L. albus* | Landrace |
| **LUP-0245** | 2 | 1.927 | -2.647 | -2.021 | *L. albus* | Landrace |
| **LUP-0248** | 4 | -3.856 | -2.844 | 1.068 | *L. angustifolius* | Wild |
| **LUP-0249** | 5 | -7.553 | -2.761 | -0.271 | *L. angustifolius* | Wild |
| **LUP-0251** | 5 | -5.331 | -0.002 | -0.105 | *L. angustifolius* | Wild |
| **LUP-0253** | 4 | -5.501 | -1.263 | 2.096 | *L. angustifolius* | Landrace |
| **LUP-0254** | 5 | -5.006 | -1.222 | -1.663 | *L. angustifolius* | Wild |
| **LUP-0255** | 4 | -3.058 | -1.531 | -1.699 | *L. angustifolius* | Wild |
| **LUP-0256** | 4 | -3.876 | -1.998 | 0.216 | *L. angustifolius* | Wild |
| **LUP-0257** | 1 | -0.552 | -1.628 | 1.071 | *L. angustifolius* | Wild |
| **LUP-0261** | 2 | 2.025 | 0.660 | 0.872 | *L. luteus* | Landrace |
| **LUP-0264** | 3 | -0.332 | 3.006 | -2.140 | *L. luteus* | Landrace |
| **LUP-0265** | 3 | -2.223 | 2.889 | -1.221 | *L. luteus* | Landrace |
| **LUP-0266** | 2 | 2.238 | 0.913 | 1.204 | *L. luteus* | Landrace |
| **LUP-0267** | 5 | -6.414 | 1.337 | 0.333 | *L. luteus* | Wild |
| **LUP-0268** | 5 | -5.019 | 1.176 | -0.269 | *L. luteus* | Landrace |
| **LUP-0269** | 2 | 2.494 | 0.611 | 0.365 | *L. luteus* | Landrace |
| **LUP-0271** | 3 | -1.418 | 3.387 | -0.616 | *L. luteus* | Landrace |
| **LUP-0273** | 3 | -0.961 | 2.402 | -1.737 | *L. luteus* | Landrace |
| **LUP-0274** | 3 | -1.691 | 4.537 | 0.530 | *L. luteus* | Wild |
| **LUP-0278** | 3 | 0.402 | 3.394 | -1.039 | *L. luteus* | Wild |
| **LUP-0279** | 2 | 3.549 | 2.138 | 2.693 | *L. luteus* | Wild |
| **LUP-0280** | 2 | 2.682 | 0.067 | 0.848 | *L. luteus* | Landrace |
| **LUP-0281** | 3 | -2.098 | 3.207 | -1.286 | *L. luteus* | Landrace |
| **LUP-0290** | 2 | 1.986 | -2.355 | -2.368 | *L. albus* | Landrace |
| **LUP-0291** | 1 | 0.538 | -1.933 | 0.786 | *L. angustifolius* | Wild |
| **LUP-0293** | 3 | 2.296 | 1.813 | -5.248 | *L. angustifolius* | Cultivar |
| **LUP-0294** | 2 | 3.862 | 4.578 | 2.603 | *L. albus* | Cultivar |
| **LUP-0295** | 1 | 0.492 | 1.853 | -0.680 | *L. luteus* | Cultivar |

Principal component scores (PC1–PC3) were obtained from a PCA performed on standardized phenotypic traits including germination, establishment, and seedling growth measured across the three experiments. Cluster membership was derived from hierarchical clustering using Euclidean distances and Ward.D2 linkage. Species identity and biological status (wild, landrace, cultivar) are shown as descriptive attributes and were not used to compute the PCA or clustering. These data summarize the multivariate phenotypic structure across accessions and support species- and status-level differentiation observed in the clustering and PCA.

**Table S18.** Performance of 48 *Lupinus* accessions under citrus-relevant substrate pH conditions and Integrated Selection Index (ISI) derived from Experiment 3 .

| **Accession** | **Cluster** |
| --- | --- |
| **LUP-0008** | 1 |
| **LUP-0020** | 5 |
| **LUP-0024** | 1 |
| **LUP-0028** | 4 |
| **LUP-0032** | 1 |
| **LUP-0074** | 2 |
| **LUP-0219** | 2 |
| **LUP-0222** | 2 |
| **LUP-0226** | 2 |
| **LUP-0227** | 2 |
| **LUP-0228** | 2 |
| **LUP-0231** | 2 |
| **LUP-0232** | 2 |
| **LUP-0233** | 2 |
| **LUP-0235** | 2 |
| **LUP-0236** | 2 |
| **LUP-0237** | 2 |
| **LUP-0238** | 2 |
| **LUP-0241** | 2 |
| **LUP-0242** | 2 |
| **LUP-0245** | 2 |
| **LUP-0248** | 4 |
| **LUP-0249** | 5 |
| **LUP-0251** | 5 |
| **LUP-0253** | 4 |
| **LUP-0254** | 5 |
| **LUP-0255** | 4 |
| **LUP-0256** | 4 |
| **LUP-0257** | 1 |
| **LUP-0261** | 2 |
| **LUP-0264** | 3 |
| **LUP-0265** | 3 |
| **LUP-0266** | 2 |
| **LUP-0267** | 5 |
| **LUP-0268** | 5 |
| **LUP-0269** | 2 |
| **LUP-0271** | 3 |
| **LUP-0273** | 3 |
| **LUP-0274** | 3 |
| **LUP-0278** | 3 |
| **LUP-0279** | 2 |
| **LUP-0280** | 2 |
| **LUP-0281** | 3 |
| **LUP-0290** | 2 |
| **LUP-0291** | 1 |
| **LUP-0293** | 3 |
| **LUP-0294** | 2 |
| **LUP-0295** | 1 |

Each accession was evaluated under two substrate pH levels representative of citrus-growing soils: acidic (pH 5.5) and neutral (pH 7.0). For each accession, the table reports establishment success, germination success, total seedling length, and hypocotyl-to-epicotyl ratio under these pH conditions, together with three normalized component scores derived exclusively from Experiment 3: Score_Establishment, Score_SeedlingLength, and Score_Stability. The Integrated Selection Index (ISI) corresponds to the mean of these three normalized components. Cluster membership is also shown for each accession as a descriptive reference and corresponds to the hierarchical phenotypic groups defined in Fig. 10A.
